# Self-sufficient biocatalytic cascade for the continuous synthesis of danshensu in flow

**DOI:** 10.1007/s00253-025-13407-3

**Published:** 2025-01-21

**Authors:** Valentina Marchini, Francesca Paradisi

**Affiliations:** 1 Department of Chemistry, Biochemistry and Pharmaceutical Sciences, University of Bern, Freiestrasse 3, 3012 Bern, Switzerland; 2 inSEIT AG, Freiestrasse 3, 3012 Bern, Switzerland

**Keywords:** Danshensu, Phenylalanine dehydrogenase, Hydroxy phenyl pyruvate reductase, Biotransformation, Enzyme immobilization, Flow reaction

## Abstract

**Abstract:**

A new strategy has been developed to successfully produce the active component danshensu ex vivo. For this purpose, phenylalanine dehydrogenase from *Bacillus sphaericus* was combined with the novel hydroxyphenylpyruvate reductase from *Mentha x piperita*, thereby providing an in situ cofactor regeneration throughout the conversion process. The purified enzymes were co-immobilized and subsequently employed in batch biotransformation, resulting in 60% conversion of 10 mM L-dopa within 24 h, with a catalytic amount of NAD^+^ as cofactor. Furthermore, the bienzymatic system was implemented as a packed-bed reactor in continuous flow, achieving a conversion rate up to 80% with 60 min retention time. The process was further intensified by implementing a 48-h flow bioreaction. The biocatalysts demonstrated remarkable stability, retaining 62% of their initial activity at the end of the process. The final productivity of the isolated compound (96% purity) was calculated to be 1.84 g L^−1^ h^−1^ yielding a sustainable synthesis of danshensu.

**Key points:**

*• Characterization of the hydroxyphenylpyruvate reductase from Mentha x piperita*

*• Bi-enzymatic system in a cascade reaction to produce danshensu*

*• Purification and isolation* *of the active compound danshensu*

**Graphical Abstract:**

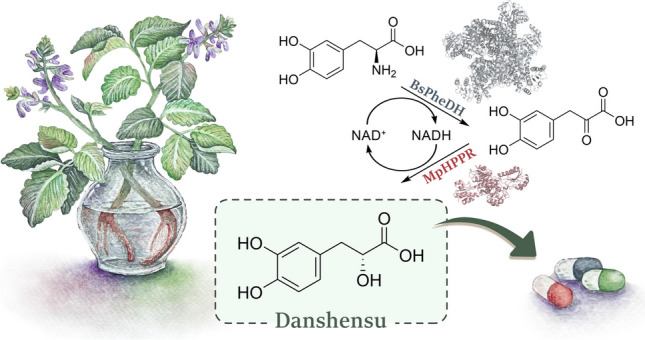

**Supplementary Information:**

The online version contains supplementary material available at 10.1007/s00253-025-13407-3.

## Introduction

The dried root of the plant *Salvia miltiorrhiza*, also known as red sage or Danshen, is one of the major traditional herb medicines in China (Li 1998). Danshen is also listed in the Chinese Pharmacopeia for its well-known pharmacological effects (Findrik et al. 2005), with the consumption of this crude drug estimated at about 80 million kilogram a year in China (Hu et al. 2005).

**Fig. 1:**
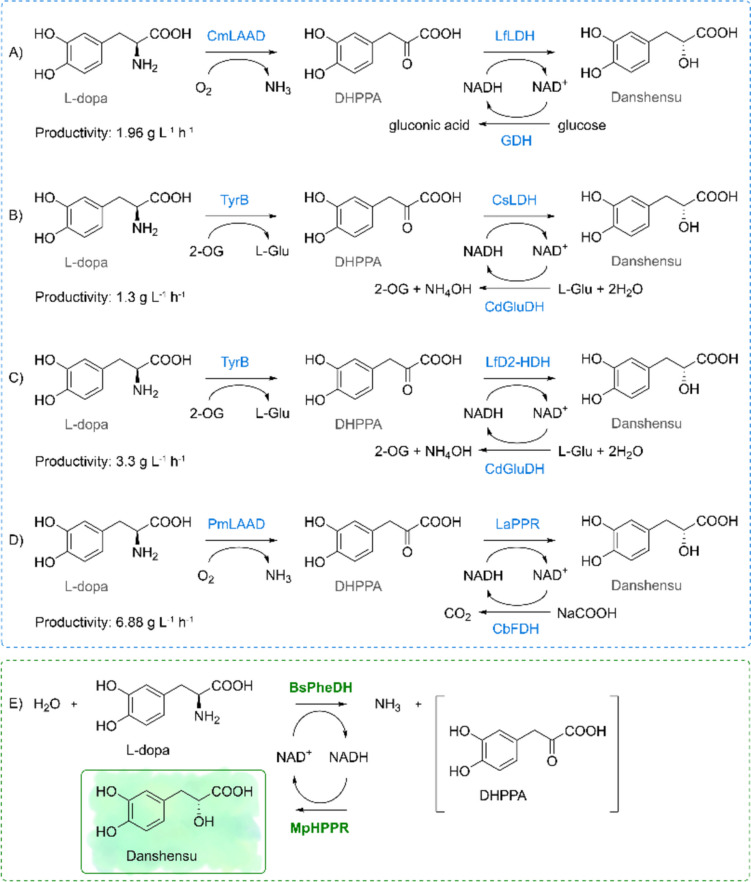
Reaction scheme of L-dopa conversion to 3,4-dihydroxyphenyl pyruvic acid (DHPPA) as intermediate and then to danshensu (*R*)-enantiomer, **A**–**D** as found in literature and **E** in our work, mediated by BsPheDH and MpHPPR. **A** CmLAAD: L-amino acid deaminase from *Cosenzaea myxofaciens*, LfLDH: D-lactate dehydrogenase from *Lactobacillus fermentum*, GDH: glucose dehydrogenase from *Bacillus* sp. G3 (Xiong et al. 2019b); **B** TyrB: transaminase from *Escherichia coli* BL21, CsLDH: D-aromatic lactate dehydrogenase from *Clostridium sporogenes*, CdGluDH: L-glutamate dehydrogenase from *Clostridium difficile*, L-Glu: L-glutamate, 2-OG: *α*-ketoglutarate (Xiong et al. 2019c); **C** LfD2-HDH: D-isomer-specific 2-hydroxyacid dehydrogenase (EC 1.1.1.345) from *Lactobacillus frumenti* (Han et al. 2023); PmLAAD: **D** L-amino acid deaminase from *Proteus mirabilis*, LaPPR: phenylpyruvate reductase from *Lactobacillus sp. CGMCC 9967*, CbFDH: formate dehydrogenase from *Candida boidinii* (Yang et al. 2023)

Danshensu [3-(3,4-dihydroxy-phenyl) lactic acid], also called Salvianic acid A, is the active component contained in Danshen. It has been extensively studied over the years, offering a list of numerous pharmacological activities (Bao et al. 2018). Danshensu is structurally composed of a catechol and a lactic acid (highlighted in green, in Fig. 1), where the catechol is the major active group that exerts an antioxidant effect (Zhang et al. 2019). In this context, it exhibited higher scavenging activity against radicals than vitamin C, in agreement with its protective effects against cell damage from oxidative stress (Zhao et al. 2008).

Danshensu demonstrated therapeutic effects in cardiovascular diseases, cerebral lesions and disorders, and other conditions such as thrombosis, tumorigenesis, pancreatitis, preeclampsia (Zhang et al. 2019). It improves blood microcirculation in association with vasodilation, hypotensive action and inhibition of platelet aggregation (Chan et al. 2004; Lam et al. 2007; Yang et al. 2010). Other mechanisms behind the beneficial effects include anti-apoptosis, inflammation regulation, lipidemia control, through several molecular signaling pathways (Kwon et al. 2014; Zhang et al. 2019, 2024; Wang et al. 2020; Hassan et al. 2024). Moreover, in vitro and in vivo studies have identified antiviral activity against severe acute respiratory syndrome coronavirus 2 (SARS-CoV-2). In parallel, danshensu effectively inhibited the related respiratory inflammation, preventing severe damage to lung tissue (Wang et al. 2022, 2024). Finally, single or repeated dose toxicity studies suggested that danshensu does not cause any sign of adverse effect and toxicity, making it a safe compound (Gao et al. 2009).

However, this promising pharmaceutical compound is too expensive for widespread commercialization. As an example, the current price of danshensu sodium salt (CAS 81075–52-7) in Sigma-Aldrich is €369 (Europe) for 25 mg (€14,760/g). Nowadays, danshensu is mostly produced by extraction from the root of Danshen, and the yield is typically low (Lam et al. 2007; Xiong et al. 2019c). Isolation and purification from natural sources are difficult and time consuming due to the chemical instability of the catechol, low content of danshensu in Danshen, and the presence of a variety of structural analogs as impurities (Dong et al. 2009). Chemical synthesis is also challenging due to its optical purity requirements (Dong et al. 2009) with positive properties established to date only for the (*R*)-enantiomer (Lu et al. 2018). Several synthetic strategies have been explored, but they suffered from complicated procedures, low yields, low enantiopurity and high costs of chiral catalysts (Yao et al. 2013; Xiong et al. 2019c). Some bioprocesses were also reported, but the employed substrates were expensive and prepared involving harsh conditions or toxic heavy metals (Yao et al. 2013; Wang et al. 2018; Ding et al. 2018; Xiong et al. 2019a, 2019c).

In the literature, four studies are reported on danshensu production starting from the renewable and low-cost substrate L-dopa, using a combination of three enzymes, as shown in Fig. 1A–D (Xiong et al. 2019c, 2019b; Han et al. 2023; Yang et al. 2023). However, a simpler two-enzyme process would reduce the overall cost, making it more sustainable.

In this work, the phenylalanine dehydrogenase from *Bacillus sphaericus* (BsPheDH, EC 1.4.1.20, AAA2264.1) was successfully coupled with a newly characterized hydroxyphenylpyruvate reductase (HPPR) to achieve an in situ cofactor recycling via hydrogen-borrowing strategy delivering a self-sufficient redox system (Fig. 1E).

## Materials and methods

### Materials

Chemicals, reagents, and medium components, unless stated otherwise, were obtained as analytical grade from Sigma-Aldrich and Fisher Scientific. Cofactors were purchased from Apollo Scientific Ltd.

3-(3,4-Dihydroxyphenyl)−2-oxopropanoic acid (DHPPA), (*R*)−3-(3,4-Dihydroxyphenyl)−2-hydroxypropanoic acid (danshensu), 3-(4-Hydroxyphenyl)−2-oxopropanoic acid (HPPA), 2-Hydroxy-3-(4-hydroxyphenyl)propanoic acid (HPLA), L-3-Phenyllactic Acid (*S*-PLA), and (R)−2-Hydroxy-3-phenylpropanoic acid (*R*-PLA) were acquired from BLD Pharma. The methacrylate resin EP400/SS was given by Resindion whereas the agarose beads were purchased by Agarose Beads Technology. The synthetic plasmid MpHPPR-pET15b was obtained from GenScript, while the plasmid BsPheDH-pTac85 was already available in house.

### Protein expression, purification, and quantification

A single colony of *Escherichia coli* BL21(DE3) Star cells previously transformed with the plasmid harboring His-BsPheDH or MpHPPR (heat-shock at 42 °C), was inoculated in 300 mL autoinduction media ZYP-5052 (1-L Erlenmeyer flasks). The cells were left to grow at 37 °C for 8 h. After 30 min of cold shock (incubation on ice and water), the flasks were left at 25 °C overnight. Cells were harvested by centrifugation at 4500 rpm (20 min, 4 °C) and resuspended in 50 mM potassium phosphate buffer, NaCl (100 mM for His-BsPheDH and 300 mM for MpHPPR) and 30 mM imidazole at pH 7.5. The suspension was placed on ice and sonicated at 50% amplitude for 8 min, with pulses of 5 s ON, 10 s OFF. After centrifugation at 14,500 rpm for 45 min, the supernatant was filtered (0.45-µm pore size) and the proteins were purified from the supernatant by a Ni–NTA column (GE Healthcare) in the ÄKTA Pure system. The proteins were eluted in 50 mM potassium phosphate buffer, NaCl (100 mM for His-BsPheDH and 300 mM for MpHPPR) and 300 mM imidazole at pH 7.5. The purified enzymes were dialyzed twice in 10 mM potassium phosphate buffer at pH 8.0 with 5 mM ß-mercaptoethanol for His-BsPheDH, 50 mM potassium phosphate buffer at pH 7.0 for MpHPPR, as described before (Asano et al. 1987; Seah et al. 1995). The concentration of MpHPPR was measured by Bradford assay. For His-BsPheDH, the following molar extinction coefficient was used: 11.7 × 10^−2^ g^−1^ mL cm^−1^. The purity of the purified proteins was evaluated by 12% (w/v) SDS-PAGE.

### Activity assay

One unit of specific activity was defined as the amount of enzyme in mg, which catalyzes the formation or depletion of 1 µmol of product, substrate or cofactor per minute. Substrates, temperature, buffer and pH value of the assay are specifically defined in Table 1 and Table S2 where activity results of each enzyme are reported. As for His-BsPheDH, the reaction mixture was prepared with 10 µL of diluted enzyme solution (500–1000-fold), 10 µL of NAD^+^ (final concentration 2.5 mM) prepared in milliQ water and 380 µL of L-Phenylalanine (10 mM as final concentration) in 100 mM KCl and 50 mM Gly-NaOH buffer pH 10.4. The activity was measured following the NADH formation at 340 nm at 25 °C. Regarding MpHPPR, the reactions were performed in a 2 mL Eppendorf by adding 10 µL of enzyme (diluted as needed), 100 µL of NAD(P)H (final concentration 10 mM) in milliQ water and 890 µL of PPA, HPPA or DHPPA as substrate (30 mM final concentration) with antioxidant (5 mM ß-mercaptoethanol or 10 mM ascorbic acid) in potassium phosphate buffer pH 7.0 (final concentration 50 mM). The reaction mixture was incubated at 37 °C, and samples were withdrawn over time to be analyzed by HPLC. The reaction product was identified and quantified through the equation obtained with a calibration curve. The kinetics constants of MpHPPR were determined by changing the substrate concentration from 0.1 to 30 mM or cofactor amount from 0.1 to 10 mM. For the oxidative reaction, 30 mM (*R*) or (*S*)-PLA were used, with 10 mM NAD^+^ and 5 mM ß-mercaptoethanol in 50 mM potassium phosphate or TrisHCl buffer at appropriate pH (incubation at 37 °C, over time). Concentrations of PPA and PLA were then analyzed by HPLC to calculate the specific activity.

**Table 1:**
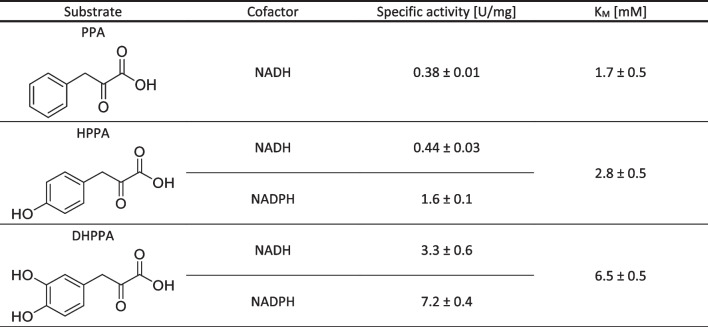
Specific activity and K_M_ for phenylpyruvic acid (PPA), 4-hydroxyphenylpyruvic acid (HPPA) and 3,4-dihydroxyphenylpyruvic acid (DHPPA) at pH 7.0 (100 mM potassium phosphate buffer) and 37 °C, using 30 mM substrate (specific activity) or different amounts from 0.5 to 30 mM (K_M_), 10 mM NAD(P)H, 5 mM ß-mercaptoethanol

### Batch biotransformations

The biocatalyst(s) were added in soluble or immobilized form to a 2-mL tube, where L-dopa or another substrate (final concentration of 10 mM, unless stated otherwise) was added together with 5 mM ascorbic acid, 1 mM NAD^+^ and 50 mM Tris HCl buffer pH 8.0. The reaction was then incubated at 37 °C with 150 rpm, if not otherwise reported. The operational stability of the immobilized biocatalysts was determined by repeating the same biotransformation multiple times (cycles), withdrawing a sample for the HPLC analysis at the end of the reaction and washing the immobilized enzymes with buffer for at least 5 times between each cycle.

### Sample preparation and analytical method

At different times, a reaction volume of 50 µL was quenched with 225 µL HCl 0.2% and 225 µL of acetonitrile. This sample was then analyzed by HPLC (Dionex UltiMate 3000 UHPLC Thermo Fisher Scientific), implemented with a C18 column (Waters X-Bridge, 3.5 µm, 2.1 × 100 mm). The flow rate was left at 0.8 mL/min and the oven was set at 45 °C. The samples were run using a gradient method from 5:95 to 95:5 (milliQ water and acetonitrile with 0.1% TFA) over 8 min. Conversions were calculated using calibration curves of authentic standards, following the production of product, and comparing it to substrate and intermediate concentrations. Residence time and wavelength of the analyzed compounds: phenyl pyruvic acid (PPA) 5.9 min at 290 nm, PLA 5.2 min at 270 nm, HPPA 5.9 min at 310 nm, HPLA 4.9 min at 280 nm, L-dopa 3.2 min at 280 nm, DHPPA 5.3 min at 310 nm, Danshensu 4.5 min at 280 nm.

### Enzyme immobilization

Typically, 1 g of support was added to 10 mL of a solution containing the desired amount of protein and incubated at room temperature under mild agitation. The protein load is defined as the amount of protein in milligrams that has been immobilized per gram of carrier (mg/g).

Small amounts of supernatant were taken over time to detect enzymatic activity, which was compared to the activity of the initial enzyme solution that was offered to the carrier (control sample). To better confirm the full immobilization, the protein concentration was then checked by Bradford assay and SDS-PAGE. The activity assay was performed by adding 5–10 mg of immobilized enzyme to a 1 ml reaction mixture containing substrates and cofactor and monitoring it using the same procedure as for the activity assay of the free enzyme. The aim is to determine the expressed activity in U/g (units of enzyme catalyzing the formation of 1 µmol of product per minute per gram of support), the specific activity in U/mg (expressed activity divided by the amount of protein loaded to the carrier) and the recovered activity, defined as a percentage calculated from the ratio between the specific activity of the immobilized enzyme and the specific activity of the free enzyme. Check Supporting Experimental Section (Supporting Information) for details on support preparation and enzyme immobilization procedures.

### Flow-reactions in a packed bed reactor

The flow reactors consisted of a R2S pumping module and a R-4 reactor heater, which were both commercially available from Vapourtec. The instrument was equipped with a glass heat exchanger assembled with an Omnifit glass column (6.6 mm bore × 150 mm length), properly filled with an appropriate amount of immobilized biocatalysts (packed bed reactor, 1.3–1.6 g). A first washing step with buffer was performed at flow rate of 0.2 mL min^−1^ to equilibrate the column. Then, a substrate solution containing 20 mM L-dopa (10 mM final concentration), 20 mM ascorbic acid and 100 mM Tris HCl buffer at pH 8.0 was pumped together with a second solution of 2 mM NAD^+^ towards the column PBR containing the biocatalyst at 0.1 mL min^−1^ for two column volumes. Afterwards, the flow rate was set up to obtain the desired residence time of the reaction. The resulting flow product was analyzed by HPLC, following the protocol described above. The product concentration is calculated as the average value of minimum 5 consecutive column volumes. Further calculations are shown in Supporting Experimental Section. The operation stability of the biocatalysts was found by leaving the flow reaction in continuous operation for a selected amount of column volumes, and comparing the final product concentration to the initial value.

## Results

### Characterization of MpHPPR

To identify a novel candidate, the sequence of HPPR from *Coleus blumei* (CbHPPR, AJ507733) was initially employed as a template in the BLAST online tool (Altschul et al. 1990). Indeed, CbHPPR was the first purified and characterized HPPR, and it accepts NADH as cofactor (required for BsPheDH activity). The HPPR from *Mentha x piperita* (MpHPPR, EC 1.1.1.237, AVZ47166.1) exhibited 91.4% identity to CbHPPR and 93% to HPPR from *Prunella vulgaris* (PvHPPR, KM053279) (Figure S1). A codon optimized synthetic gene inserted in pET15b was acquired from GenScript (Figure S3) and the enzyme was successfully expressed and purified with the His-tag at the N-term (Figure S4 and S5).

The biocatalyst demonstrated exclusive activity with the (*R*)-enantiomer of phenyl lactic acid (PLA), used as test substrate, and the biocatalyst highly favored the reduction of phenylpyruvate over the oxidation of *R*-PLA (reverse reaction), with a 1000-fold difference in activity (Figure S6). Dual cofactor specificity was also observed, albeit with a preference for the phosphorylated cofactor. pH 7.0 and 37 °C were chosen as optimal standard conditions for the activity assays (Figure S7). The specific activities of MpHPPR for the different substrates and cofactors are reported in Table 1 (see Table S1 for activities with different antioxidants).

### Specific activity of His-BsPheDH

BsPheDH was originally cloned into pTac-85 vector; however, protein precipitation by addition of 60% saturated ammonium sulfate resulted in incomplete purification (Asano et al. 1987). Consequently, to facilitate the enzyme purification process, a poly-histidine tag was added to the protein N-terminus by subcloning the BsPheDH gene into pRSETb vector (Figure S2). The specific activity of His-BsPheDH towards L-phenylalanine at pH 10.4 and 25 °C was 120 ± 9 U/mg in the oxidative deamination reaction, highly similar to the values reported in the literature for BsPheDH (Asano et al. 1987; Seah et al. 1995; Khorsand et al. 2017), therefore the enzyme activity was not affected by the His-tag addition. His-BsPheDH demonstrated lower activity at pH values below 8.5, with residual activity levels of less than 20% (Table S2). At higher temperatures of 30 °C and 37 °C, the specific activity exhibited a slight increase, reaching 1.1-fold and 1.21-fold, respectively.

### Combination of His-BsPheDH and MpHPPR (soluble enzymes)

**Fig. 2:**
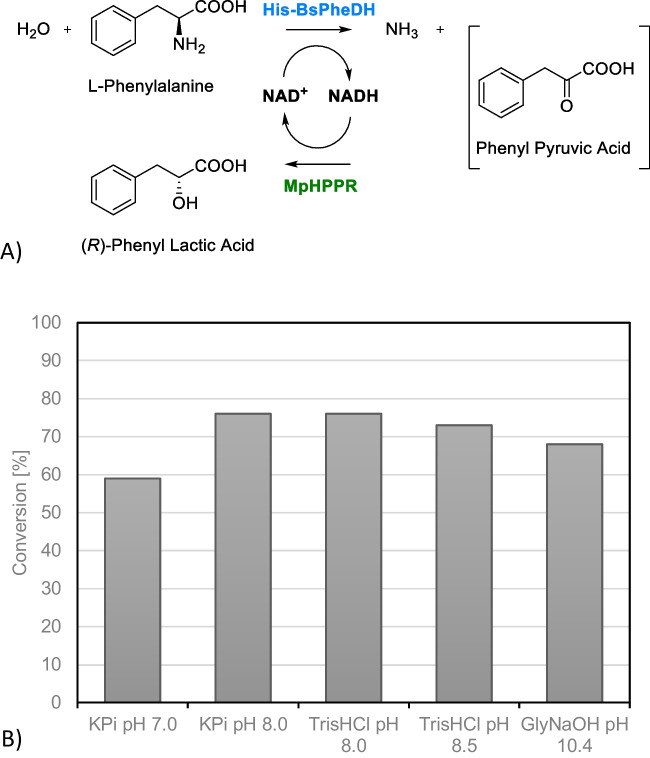
**A** Reaction scheme and **B** degree of conversion after 2 h of biotransformations using different buffers and pH, with 5 mM L-phenylalanine, 10 mM ascorbic acid, 1 mM NAD^+^ in 1 mL total volume, at 37 °C with 150 rpm of shaking. Enzymes concentrations: 0.18 mg/mL His-BsPheDH and 0.45 mg/mL MpHPPR

The optimal temperature was determined to be 37 °C in both cases, and ascorbic acid was identified as the most effective antioxidant to retain high activity. However, the pH preference varied significantly between the two catalysts; MpHPPR demonstrated a clear preference for pH 7, while His-BsPheDH exhibited a clear preference for pH 10.4. pH 8 was chosen as a compromise showing sufficient activity of both enzymes while simultaneously reducing the oxidation rate of the substrate, which is known to increase with rising pH values. To ensure a comprehensive evaluation, using L-phenyl alanine as a test substrate, the cascade reaction was tested with different buffers (Fig. 2). The conversion of L-phenylalanine to PLA reached 76% in TrisHCl and potassium phosphate (KPi) at pH 8 (Fig. 2,B), however, in KPi, L-dopa was noted to oxidize more rapidly (results not shown) and 50 mM Tris–HCl, pH 8.0, with 10 mM ascorbic acid at 37 °C was selected as the optimal medium.

**Fig. 3:**
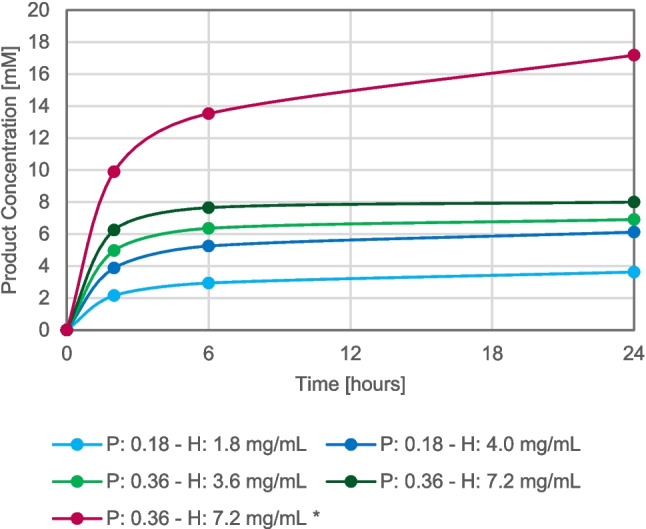
Product formation over time of biotransformation, employing different concentrations of biocatalysts (P: His-BsPheDH—H: MpHPPR). Reaction conditions: 10 mM L-dopa (* 50 mg/mL of L-dopa suspension), 10 mM ascorbic acid, 1 mM NAD^+^ in 50 mM TrisHCl pH 8.0. Total volume: 1 mL. Incubation at 37 °C and 150 rpm. Samples are measured in triplicate

Under the selected conditions, the specific activity of His-BsPheDH with L-dopa was determined to be 10.6-fold higher than that of MpHPPR with DHPPA (18 ± 1 U/mg and 1.7 ± 0.3 U/mg, respectively), and this was used as guidance to the optimization of the cascade reaction with L-dopa (Fig. 3).

A combination of 0.36 mg/mL of His-BsPheDH and 7.2 mg/mL of MpHPPR (in a ratio of 1:2 U with exactly 6.5 and 13 units), yielded an 80% conversion into danshensu in 24 h at a 10 mM scale (2 mg/mL). The solubility of L-dopa is very low (5 mg/mL); however, to increase the reaction productivity, L-dopa can be prepared as a suspension. Therefore, in a parallel biotransformation, a 50 mg/mL L-dopa suspension was tested, leading to a notable increase in product formation, reaching 17.2 mM in 24 h, more than double of what could be achieved with a soluble feedstock.

### Immobilization screening

The two biocatalysts were immobilized on the methacrylate resin EP400/SS using different strategies (see paragraph 5, SI), following in silico analysis of the sequences with CapiPy (Roura Padrosa et al. 2021; Marchini et al. 2021) (Figures S8-S9). This support demonstrated complete immobilization yields. His-BsPheDH showed 36% recovered activity when covalently immobilized on epoxy/cobalt, and 92% when ionically anchored to the support following polyethyleneimine (PEI) coating, with a protein loading of 1 mg/g (Table S3). Similarly, MpHPPR recovered 39% of activity with covalent chemistry, and 89% on PEI, with a protein loading of 5 mg/g (Table S4).

His-BsPheDH and MpHPPR were then co-immobilized on EP400/SS testing ionic interactions (amino groups), covalent binding (epoxy/cobalt), or a combination of both (His-BsPheDH on aldehydes and MpHPPR on amino groups, Figure S10). This latter strategy, albeit with different chemistries, has been previously described for the immobilization of other enzymes (Velasco-Lozano et al. 2017, 2018). A loading of 5 mg/g of His-BsPheDH and 40 mg/g of MpHPPR was used to ensure rapid conversion. Complete immobilization was obtained and biotransformations were conducted to evaluate the performance of the co-immobilized enzymes and their operational stability. The immobilized biocatalysts were subjected to six cycles of short biotransformations, with each cycle comprising a two-hour reaction. Covalent immobilization exhibited the greatest stability with no loss of activity (Figure S11).

The co-immobilization was then extensively optimized (paragraph 6, SI) to maximize both the immobilized activity and the operational stability, including also the more hydrophilic agarose support which indeed showed better performance (Tables S6 and S7). Eventually, the sequential co-immobilization on epoxy/cobalt agarose with the initial addition of 40 mg/g MpHPPR for 24 h and then 2.5 mg/g of His-BsPheDH for an additional day showed the highest product formation in 24-h biotransformations, specifically 4.9 mM (Figure S12). Moreover, the same strategy reached the best operational stability after three cycles of 24-h biotransformations, retaining 70% of its initial activity (Figure S13). The substitution of glycine with ethylenediamine (EDA) as blocking agent, to block the unreacted groups on the support, led to a notable enhancement in both product formation and the operational stability achieving 6 mM danshensu in 24 h and the retention of 75% activity after three consecutive cycles of reactions involving 5 mg/g His-BsPheDH and 40 mg/g MpHPPR (Figure S14). This strategy outperformed both mixing of individually immobilized enzymes (separate resins), and alternative co-immobilization approaches (Figure S12).

### Batch biotransformation of free and co-immobilized enzymes

**Fig. 4:**
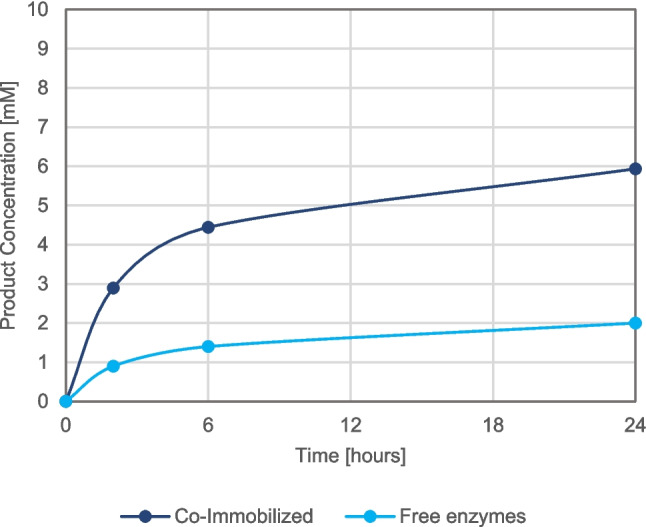
Product formation over time of biotransformation, employing same units of activity but different enzymes forms: 0.3 U of His-BsPheDH—3 U of MpHPPR corresponding to 100 mg of co-immobilized enzyme on agarose with epoxy/cobalt—EDA as blocking agent, 0.017 mg/mL of free His-BsPheDH with 1.8 mg/mL of free MpHPPR. Reaction conditions: 10 mM L-dopa, 10 mM ascorbic acid, 1 mM NAD^+^ in 50 mM TrisHCl pH 8.0. Total volume: 1 mL. Incubation at 37 °C and 150 rpm. Samples are measured in triplicate

The free and co-immobilized biocatalysts were evaluated in batch biotransformations of L-dopa, with equal amounts of units added, specifically 0.3 U of His-BsPheDH and 3.0 U of MpHPPR in both preparations (Fig. 4). For the co-immobilized enzyme preparation, the specific activity of each enzyme (U) was extrapolated from the tests performed on the individually immobilized biocatalysts. Despite the higher initial activity of the free enzymes, the immobilized version demonstrated superior performance. After 24 h, the concentration of danshensu reached up to 6 mM for the co-immobilized biocatalysts and only 2 mM for the free form.

### Biosynthesis of danshensu in continuous flow

To drastically decrease the reaction time, the biotransformation was performed in continuous flow. The faster reaction time was also expected to reduce the rate of oxidation of L-dopa or DHPPA.

Optimal co-factor concentration was established at 1 mM, with no improvement on conversion when 2 mM was adopted and lower conversions when only 0.1 mM of NAD^+^ was utilized.

The packed-bed reactor was filled with 1 g of co-immobilized His-BsPheDH (5 mg/g) and MpHPPR (40 mg/g) (Table 2).

**Table 2:**
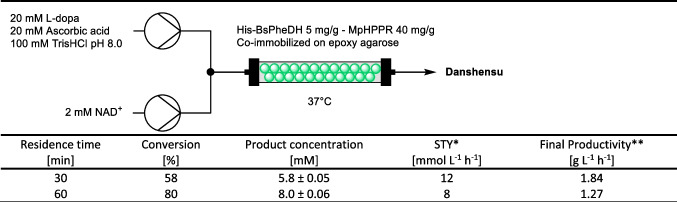
Set-up of the flow reactor for the continuous conversion of L-dopa to danshensu and related results

^*^Volume PBR: 1.9 mL. Flow rate: 0.064 mL/min (R_t_: 30 min), 0.032 mL/min (R_t_: 60 min)

^**^MW danshensu: 198.17 g/mol. Extraction yield: 80%

Following a 30-min residence time, L-dopa was converted to 5.8 mM of danshensu. Compared to the batch biotransformation, the reaction time has efficiently decreased from 24 h to 30 min to produce the same amount of danshensu (approximately 6 mM).

An increase in the residence time to 60 min resulted in an enhanced conversion, reaching 80% with 8.0 mM of produced danshensu per hour at 37 °C (see HPLC calibration curves and spectra in Figures S16 and S17). The absence of intermediate products throughout the course of the reaction indicates that MpHPPR is not a limiting factor in the overall conversion process.

The system was evaluated over 24 and 48 h of continuous flow reaction to assess the operational stability of the catalytic resin. Independently of the residence time, the retained activity was 75% after 24 h and 62% after 48 h, indicating that the system remained stable over the course of the reaction.

Danshensu was reported to be extractable in ethyl acetate after acidification achieving 80% yield (Zhang et al. 2008). The downstream solution collected from the flow reactor was thus extracted in EtOAc after acidification, dried and analyzed by ^1^H-NMR (Figures S19). A preliminary test of continuous flow reaction was performed with β-mercaptoethanol as antioxidant which resulted in low purity of the desired compound, since β-mercaptoethanol was extracted with danshensu (Figure S18). Therefore, ascorbic acid was chosen as antioxidant agent in all the biotransformations.

The ^1^H-NMR spectra confirmed the presence of danshensu (96% purity) with ascorbic acid as an impurity at a concentration of 4%. Remarkably, the compound was not detected in the oxidized form. The final yield of danshensu resulted in 46% with 30-min residence time and 64% with 60 min.

**Fig. 5:**
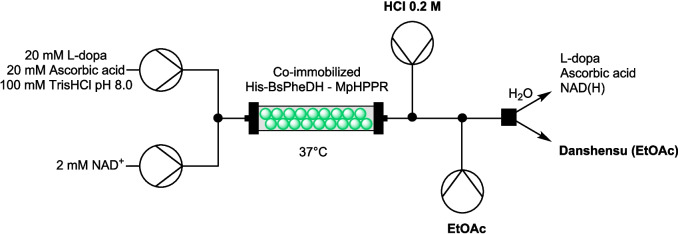
Second set-up of the flow reactor for the continuous conversion of L-dopa to danshensu and in-line product separation

A second set-up (Fig. 5) was trialed to test whether danshensu could be extracted in continuous. A 0.2 M HCl solution inlet was mounted downstream of the packed-bed reactor (flow rate: 0.015 mL/min), followed by an inlet of ethyl acetate (flow rate 0.1 mL/min). A Zaiput liquid–liquid separator was added to separate the phases. Danshensu could be effectively extracted in the solvent phase; however, the extraction yield decreased to approximately 60%.

## Discussion

### Selection and characterization of a novel HPPR

HPPRs are a group of enzymes belonging to the class of oxidoreductases and, more specifically, to the family of D-isomer-specific 2-hydroxyacid dehydrogenases (Janiak et al. 2010). To date, only two HPPRs have been recombinantly expressed, the HPPR from *Coleus blumei* (CbHPPR) and the HPPR from *Prunella vulgaris* (PvHPPR) which are both NAD(P)H dependent (Kim et al. 2004). Two additional enzymes belonging to this group have been characterized, originating from *Salvia miltiorrhiza* and from *Arabidopsis thaliana* (Wang et al. 2017; Xu et al. 2018), but as they are NADPH selective they were not considered, due to the strict dependence of BsPheDH on NADH (Noriko et al. 1988).

A comparative analysis of the amino acid sequences of MpHPPR and CbHPPR revealed the presence of two residues exhibiting distinct characteristics within the active site region. Indeed, the serine and asparagine in positions 53 and 54, respectively, of CbHPPR are alanine and threonine in MpHPPR. It was postulated that the residue at position 52 (asparagine) would interact with the para-hydroxy group of the substrate, as observed for the CbHPPR (Janiak et al. 2010). It can thus be suggested that the connected positions 53 and 54 may be involved in the formation of the pocket shape, in which the aromatic moiety of the substrate is inserted. Given that these different amino acids have undergone a change in spatial distribution and side groups, it is possible that the two enzymes may differ in terms of their activity and substrate specificity, despite the high degree of identity. In particular, the side chain of Ser53 is a hydroxyl group (Figure S1) that may reduce the available space and interfere with the interaction of the substrate hydroxyl groups. In contrast, the alanine at position 53 of MpHPPR allows for greater space and interaction with the catechol. The other characterized enzyme, PvHPPR, possessed alanine at position 53 and threonine at position 54, similar to MpHPPR. However, the activity towards DHPPA was not reported (Ru et al. 2023).

With specific activities between 3 and 7 U/mg, MpHPPR was found to be 100 times more active than CbHPPR with the target substrate DHPPA (0.028 U/mg with NADH and 0.072 U/mg with NADPH), and a minimal difference between MpHPPR and PvHPPR was observed towards HPPA and NADH (0.44 and 0.63 U/mg, respectively), while CbHPPR activity was as low as 0.01 U/mg with this latter substrate (Kim et al. 2004; Ru et al. 2023). The reduced activity of CbHPPR in comparison to the two other proteins may be indicative of a difference in the spatial configuration of the active site, which is influenced by the specific amino acid residues present at positions 53 and 54.

CbHPPR showed maximal activity at 37 ℃ and at pH of 6.5 to 7.0 with HPPA or DHPPA as substrates, comparable to the results obtained with MpHPPR (Häusler et al. 1991).

MpHPPR showed a preference for NADPH as cofactor, exhibiting nearly fourfold higher specific activity with HPPA as a substrate and more than double the activity with DHPPA compared to that achieved with NADH. A similar pattern was described for CbHPPR. However, CbHPPR does not accept PPA as a substrate, as the reduction occurred at a negligible rate, less than 2% of the activity observed with DHPPA (Kim et al. 2004). Conversely, MpHPPR exhibited notable activity towards PPA, comparable to its specific activity towards HPPA.

Of the three tested substrates, MpHPPR demonstrated a clear preference for DHPPA, while CbHPPR had the highest activity towards PPA (Häusler et al. 1991). Indeed, the kinetic measurements revealed an increase in the values with the addition of hydroxy groups on the aromatic moiety which correlates with an increase in steric hindrance as well as polarity.

MpHPPR accepted DHPPA and NADH as substrates, thus providing a promising candidate for further study and optimization within a biocatalytic cascade for danshensu production.

### His-BsPheDH and MpHPPR cascade (free form)

Activity conditions were screened in order to find a compromise between the two enzymes.

To ensure comparable units of activity (ratio 1:1), the concentration of MpHPPR had to be approximately 10 times higher than PheDH (1:10 mg/mL of His-BsPheDH and MpHPPR). Ratios of 1:1 and 1:2 in units of activity were evaluated in the biotransformations with L-dopa (Fig. 3). An increase in enzyme concentration and unit ratio resulted in a slight enhancement in conversion rate; however, the maximum concentration of His-BsPheDH that could be tested was 0.36 mg/mL, due to the low expression levels. In contrast, MpHPPR demonstrated no particular limitations.

A parallel biotransformation was performed with a suspension of L-dopa at 50 mg/mL. Indeed, the maximum solubility of this compound is reported to be 5 mg/mL in water at 20 °C (~ 25 mM) (Dannenfelser and Yalkowsky 1991). Through prolonged sonication, we were able to reach 30 mM of L-dopa at pH 8 and 40 °C. In this instance, the inclusion of ascorbic acid as an antioxidant was crucial to maintain the stability of L-dopa, which rapidly oxidizes when exposed to moisture and air, at alkaline pH, and high temperatures, forming a visible dark precipitate of melanin (Siddhuraju and Becker 2001; Zhou et al. 2012). The increase from 10 mM (approximately 2 mg/mL) to 50 mg/mL, resulted in enhanced conversion rates, with the danshensu concentration doubling within a 24-h period. The substrate suspension was readily solubilized in the reaction environment to maintain a constant concentration of L-dopa, up to the maximum solubility, while the conversion proceeded. This promoted the progression of the cascade reaction towards the product formation with a productivity of 0.142 g L^−1^ h^−1^, but the free form of the biocatalysts did not allow for their reuse and separation, as the immobilized form might offer.

Previous publications have reported the biocatalytic production of danshensu starting from L-dopa, in whole cell biotransformations using three concatenated enzymes, wherein the third biocatalyst was essential for the continuous regeneration of the cofactor (Xiong et al. 2019c, 2019b; Han et al. 2023; Yang et al. 2023). In our study, only two enzymes were employed, and the cofactor was directly recycled in situ via the double redox mechanism. Therefore, the costs associated with the enzymes and related substrates, as well as the time required for the biocatalysts preparation are reduced.

It must be noted that the first two published processes (Fig. 1A, B) declared 1.96 and 1.3 g L^−1^ h^−1^ of crude productivity (Xiong et al. 2019c, 2019b) with a stated initial concentration of 125 mM of L-dopa (pH 7.5, 35 °C) and 119 mM of (*R*)-danshensu produced. In our opinion this appears questionable due to the certified low solubility of the substrate (around 25 mM) and product (20 mg/mL, 100.9 mM). Given that the reaction was conducted at 35 °C for 12 or 18 h in the absence of an antioxidant, it is highly likely that the substrate and intermediate were oxidized, as was briefly reported in one of the two papers. Therefore, the stated productivity is likely grossly overestimated.

Han et al. also (Fig. 1C) reported concentrations of L-dopa ranging from 20 to 100 mM at 35 °C and pH 7 in the absence of an antioxidant (Han et al. 2023). A 40 mM L-dopa solution yielded 33.5 mM of danshensu in 2 h at a 10-mL scale, with a productivity of 6.61 g L^−1^ h^−1^. However, our calculations would give a value of 3.3 g L^−1^ h^−1^ at best and, as mentioned previously, the value is not entirely reliable. Following a 2-h period in the absence of an antioxidant, the author observed that the concentration of danshensu declines gradually as the reaction proceeds, accompanied by a gradual accumulation of by-products (oxidized substrate and intermediate, probably), particularly after 9 h. It does not appear possible to reliably assess these reactions without an antioxidant to prevent substrate/intermediate/product oxidation.

Finally, the work reported by Yang and coworkers (Fig. 1D) should be considered (Yang et al. 2023). In their paper, the activity assay of L-amino acid deaminase from *Proteus mirabilis* (PmLAAD) was conducted with 20 mg/mL of L-dopa and measured by UV which would give light scattering due to the presence of insoluble solid particles, possibly interfering with the assay. The whole-cell biotransformation yielded 82.6 g/L of danshensu (from 90 g/L of L-dopa as fed-batch system, 30 °C, pH 7) in 12 h, at 100-mL scale. Sodium formate (1.6 M) was added at a concentration of 108 g/L, resulting in the accumulation of ammonia as a byproduct (up to 456 mM). Both factors accelerate the oxidation process. The productivity could be calculated at 6.88 g L^−1^ h^−1^ but, again, the maximum solubility of danshensu is around 20 g/L, which is four times lower than what the authors reported. In enzymatic reactions, and particularly in whole-cell systems, the final concentration of a product cannot exceed the maximum solubility in the reaction environment. Without employing an alternative strategy to achieve a higher product concentration, such as biphasic solvent systems, it is highly unlikely to achieve a quantity of product that is four times its maximum solubility. Moreover, the HPLC analysis, which monitors the reaction, is conducted by first centrifuging the reaction components and then injecting the supernatant into the HPLC system. Consequently, only the soluble components are visible in the chromatogram, whereas the insoluble components, including both the substrate and the product, are not present. It is unclear how the final 82.6 g/L of product were weighed or calculated to assess the final productivity, considering both the HPLC analysis limit and the maximum solubility issue.

Furthermore, it is crucial to acknowledge that in all four published works, the product was not subjected to purification processes, and the amount of danshensu was merely estimated based on its concentration in the resulting solution, together with all the other reaction components and by-products.

### Immobilization of MpHPPR and His-BsPheDH, and batch biotransformations

To increase the enzyme stability and enhance the reusability, the biocatalysts were immobilized following the screening of selected conditions and the evaluation of different supports.

The optimization step resulted in a significant enhancement in the immobilization of MpHPPR. A prolongation of the incubation period from 8 to 24 h resulted in a recovery of nearly twice the initial activity. Given the high protein concentrations initially provided (40 mg/g), a longer period was necessary to ensure that all the dimeric structures were fully covalently bound.

Despite the optimization process, the recovered activity of His-BsPheDH did not exceed 8% when 5 mg/g were loaded which was attributed to the requirement of β-mercaptoethanol in the protein solution (Table S5) (Asano et al. 1987).

It has been previously documented that β-mercaptoethanol can interact with epoxides through nucleophilic attack and it has in fact been employed as blocking agent in immobilization (Mateo et al. 2000). If β-mercaptoethanol blocks the reactive groups, these are less available for the enzyme to bind covalently, that is then bound to the support through unspecific weak interactions, causing protein leaching over time. This behavior was confirmed by running samples of boiled resin on an SDS-PAGE (see Figure S15). The leaching effect was found to be significantly reduced when incubation was prolonged to 24 h and EDA was used as a stabilizing agent, although not entirely eliminated. Given the conformation of His-BsPheDH, it can be postulated that a longer binding time is required for all eight subunits to effectively interact with the epoxides on the support, particularly when fewer functional groups are available.

Due to the low expression levels and purification yields, the small concentrations of His-BsPheDH were predominantly immobilized, as they were obtained after dialysis with minimal dilution. It can be reasonably assumed that higher enzyme concentrations with a greater number of resulting dilutions would result in a reduction of the β-mercaptoethanol concentration, and therefore a decrease in its adverse effects on His-BsPheDH immobilization.

The co-immobilized enzymes were evaluated in the two step biotransformations. At equivalent conditions of immobilization, the co-immobilized enzymes exhibited superior performance to the singularly immobilized proteins, with a conversion rate exceeding twofold after 24 h of biotransformation. Indeed, the closer proximity of His-BsPheDH and MpHPPR active sites facilitates and accelerates the exchange of DHPPA and co-factor through tunneling effect (Marchini et al. 2021, 2022).

Furthermore, the sequential co-immobilization approach, initiated with MpHPPR, demonstrated the most favorable outcomes, providing additional evidence for the detrimental impact of β-mercaptoethanol. When His-BsPheDH was incubated as the final step, the number of reactive groups exhibited a decline solely during its immobilization, without influencing the preceding MpHPPR incubation phase. The polar amino groups of EDA then served as stabilizers on the support, enhancing the operational stability of the two biocatalysts over repeated cycles of usage.

Finally, the co-immobilized biocatalysts exhibited a consistent threefold increase in conversion compared to the free enzymes at all time points (2, 6, and 24 h). Consequently, the co-immobilization process proved an effective means of enhancing the performance and stability of the two proteins under biotransformation conditions. In 24 h, 60% of L-dopa was successfully converted into danshensu with the co-immobilized enzymes, using only 0.3 Units of His-BsPheDH and 1 mM of cofactor (the ratio of NAD^+^ and L-dopa is 1:10).

### Biosynthesis of danshensu in continuous flow

This work demonstrates the effective continuous production of danshensu in a sustainable manner. Furthermore, the co-immobilized enzymes exhibited good stability across different column volumes of flow reactions. The final productivity of the isolated danshensu resulted in 1.84 g L^−1^ h^−1^. Although previous reports have indicated productivities as high as 7 g L^−1^ h^−1^ (albeit possibly overestimated as discussed above) this represents the first continuous production of danshensu with immobilized enzymes, having work-up procedure. In this study, the two biocatalysts can be readily separated from the reaction environment, allowing for their reuse in multiple cycles of danshensu production. In comparison, the previously reported whole-cell biotransformations were limited to a single cycle, as the biocatalysts could not be recovered after the 2–18 h reaction.

In contrast, the cell-free His-BsPheDH–MpHPPR biotransformation system demonstrated sustained activity and stability over a 2-day period. It is conceivable that the immobilized biocatalysts could be continuously utilized for longer periods to sustain the danshensu production process, obviating the necessity for novel enzyme expression. This work has the potential to be implemented in an industrial setting as a highly sustainable process. The system can be implemented with an in-line separation process consisting of an HCl stream (acidification) followed by a flow of ethyl acetate (solvent extraction) to collect danshensu in the solvent phase, as demonstrated as a proof of concept. Conversely, the water phase containing unreacted substrates and unextracted danshensu may be potentially recirculated into the system after proper pH adjustment, thus creating a closed-loop sustainable process that prevents solvent waste. With the implementation of appropriate optimization techniques, the automation of work-up procedures can be efficiently incorporated into the system. This has the potential to reduce the time and cost associated with the process, while also minimizing the environmental impact.

The novel HPPR from *Mentha x piperita* has been successfully expressed and characterized, demonstrating activity in the reduction of DHPPA with NADH. This enzyme was effectively coupled to His-BsPheDH for the production of danshensu. A fully optimized reusable system was obtained after co-immobilization of the two enzymes on agarose. Their implementation in a flow bioreactor offered a 58% conversion with a 30-min residence time and 80% conversion with 60 min. Subsequently, the two biocatalysts were evaluated for their performance in a continuous flow biotransformation over 24 and 48 h, exhibiting remarkable stability and reusability across multiple reaction cycles. A productivity of 1.84 g L^−1^ h^−1^ was achieved. The solution was collected, extracted, and the product was further characterized to effectively confirm the structure and purity of the final product, which was not reported before.

Complete conversion was probably not achieved since the two enzymes work best at different pH values. In fact, His-BsPheDH works most efficiently at pH 10.4, which would oxidize L-dopa in a short time despite the presence of antioxidant, while MpHPPR prefers pH 7, which is too low for the former enzyme. The chosen conditions were a compromise to have the highest conversion with the coupled bienzymatic system.

The production of danshensu was successfully achieved in a sustainable manner. This may facilitate the development of cost-effective synthesis methods and, subsequently, expanded pharmacological applications, which were previously not considered due to the high price of danshensu.

## Supplementary Information

Below is the link to the electronic supplementary material.

Supplementary file1 (PDF 2001 KB)

## Data Availability

The authors declare that the data supporting the findings of this study are available within the paper and its Supplementary Information file. Should any raw data files be needed in another format they are available from the corresponding author upon reasonable request.
