# Supplementary material for: Self-sufficient biocatalytic cascade for the continuous synthesis of danshensu in flow
Source: Appl Microbiol Biotechnol. 2025 Jan 21;109(1):13. doi: 10.1007/s00253-025-13407-3 (PMC11750938; doi:10.1007/s00253-025-13407-3)
Supplement: Supplementary file 1 — Supplementary file1 (PDF 2001 KB) [file 253_2025_13407_MOESM1_ESM.pdf]

Supplementary Information

Applied Microbiology and Biotechnology

Self-sufficient biocatalytic cascade for the continuous synthesis of danshensu in flow

Valentina Marchini [1,2] and Francesca Paradisi *[1]

1 Dr. V. Marchini (ORCID: 0000-0002-0294-166X), Prof. Dr. F. Paradisi (ORCID: 0000-0003-1704-0642)
Department of Chemistry, Biochemistry and Pharmaceutical Sciences
University of Bern
Freiestrasse 3, 3012 Bern (Switzerland)
E-mail: francesca.paradisi@unibe.ch

2 inSEIT AG
Freiestrasse 3, 3012 Bern (Switzerland)

Table of Content

SUPPORTING EXPERIMENTAL SECTION 3

Activation of supports and enzyme immobilization 3

Continuous Flow Reaction – Calculations 3

SUPPORTING RESULTS SECTION 4

1. Sequence alignment of HPPRs 4
2. Expression and purification of MpHPPR 6
3. Characterization of MpHPPR 8
4. Characterization of His-BsPheDH 9
5. Immobilization screening of His-BsPheDH and MpHPPR 10
6. Optimization of protein immobilization 12
7. Characterization of danshensu 16
8. Supplementary References 20

SUPPORTING EXPERIMENTAL SECTION

Activation of supports and enzyme immobilization

The resin was left in incubation with the enzyme solubilized in the appropriate buffer. All the procedures are done at room temperature, if not otherwise stated, under mild agitation. The washing steps are performed with deionized water or appropriate buffer. All the resins are stored at 4°C after preparation or immobilization.

For the activation of epoxy resin to aldehyde groups, 1 g of support was mixed with 10 mL of 100 mM H_2SO_4 and incubated overnight. The support was washed, incubated with 10 ml of 30 mM $NaIO_4$ for 2 h and washed again. For the immobilization, the protein solution was prepared in 100 mM $NaHCO_3$ buffer pH 10. The support was then washed, and the Schiff bases were reduced by incubating the support with 10 mg of $NaBH_4$ in 10 mL of 100 mM $NaHCO_3$ pH 10 for 30 min at 4°C (Guisán 1988).

For the covalent immobilization on epoxy resin by first interaction with a metal, 1 g of resin was incubated with 2 mL of modification buffer (100 mM sodium borate, 2 M iminodiacetic acid, 50 mM phosphate buffer pH 8) for 2 h. After washing, the support was incubated with 5 mL of metal buffer (1 M NaCl, 5 mg/mL $CoCl_2$ in 50 mM phosphate buffer pH 6) for 2 h. The support was then washed and incubated with the protein solution in phosphate buffer 50 mM pH 8.5. The support was then washed with 3 mL of desorption buffer (50 mM EDTA, 500 mM NaCl in 50 mM phosphate buffer pH 7) to remove the metal and rinsed with deionized water. The support was left in 4 mL blocking buffer (3 M glycine in 20 mM phosphate buffer pH 8.5) for an overnight and then washed (Mateo et al. 2007).

For the covalent immobilization on epoxy groups by interaction with ethylenediamine (EDA), 1 g of the epoxy resin was incubated with 6 mL of 2% (v/v) EDA in 100 mM $NaHCO_3$ buffer pH 8.5 for 2 h. The support was then washed (water, 1 M NaCl and again with water). The protein solution was prepared in 5 mM phosphate buffer pH 7. After being washed, the immobilized enzyme was incubated with 1 mL of 5 mM $NaHCO_3$ buffer pH 9 for at least 2 h. Afterwards, the support was washed and left in 4 mL blocking buffer (prepared as previously described) for an overnight (Trobo-Maseda et al. 2020).

For the coating of glyoxyl resin with polyethyleneimine (PEI), a solution of 10 mg/mL of PEI 60 kDa was prepared in 100 mM $NaHCO_3$ buffer pH 10 and 10 mL were incubated with 1 g of support for an overnight. After that, the epoxy resin was washed and stored, while the glyoxyl resin was both washed and incubated with 1 mg of $NaBH_4$ in 10 mL of 100 mM $NaHCO_3$ buffer at pH 10 for 30 min at 4°C. The protein solution was prepared in 5 mM phosphate buffer pH 7. After immobilization, the resin was washed and stored (Mateo et al. 2000; Velasco-Lozano et al. 2017).

Continuous Flow Reaction – Calculations

Reactor volume [mL]: PBR column length [cm] x 0.3421

Flow rate [mL/min]: Reactor volume [mL] / Residence Time [min]

Flow rate [L/h]: Flow rate [mL/min] x 60 x 10^{-3}

Space-Time Yield (STY) [mol L^{-1} h^{-1}]: Flow rate [L/h] x Product concentration [M] / Reactor volume [L]

Final Productivity [g L^{-1} h^{-1}]: STY [mol L^{-1} h^{-1}] x MW product [g mol^{-1}] x Extraction Yield [%] x Purity [%]

SUPPORTING RESULTS SECTION

1. Sequence alignment of HPPRs

```
CbHPPR    MEAIGVLMMCPMSTYLEQELDKRFKLFRYWTQPAQRDFLALQAESIRAVVGNSNAGADAE 60
MpHPPR    MEAIGVLMTCPMNNYLEQELDKRFKLFRYWTQPKQREFLAQHAESIRAVVGNATAGADAE 60
PvHPPR    MDAIGVLMMCPMSNYLEQELDNRFKLFRYWAQPKQRDFLADHAASIRAVVGNATAGADAE 60
          *:****** ***..*******:********:** **:*** :* ********:.******

CbHPPR    LIDALPKLEIVSSFSVGLDKVDLIKCEEKGVRVTNTPDVLTDDVADLAIGLILAVLRRIC 120
MpHPPR    LIDALPKLEIVSSFSVGLDKVDLNKCKEKGIRVSNTPDVLTDDVADLAIGLMLAVLRRIC 120
PvHPPR    LIASLPKLEIVSCFSVGLDKVDLIKCKEKGIRVTNTPDVLTDDVADLAIGLILAVLRRIC 120
          ** :********.********** **:***:**:*****************:********

CbHPPR    ECDKYVRRGAWKFGDFKLTTKFSGKRVGIIGLGRIGLAVAERAEAFDCPISYFSRSKKPN 180
MpHPPR    ECDKYVRRGAWKLGDFKLTTKFSGKRVGILGLGRIGLAVAERAEAFDCPISYYSRSKKAN 180
PvHPPR    ECDKYVRRGAWKLGDFKLTTKFSGKRVGIIGLGRIGLAVAERAEAFDCPISYYSRSKKGN 180
          ************:****************:**********************:***** *

CbHPPR    TNYTYYGSVVELASNSDILVVACPLTPETTHIINREVIDALGPKGVLINIGRGPHVDEPE 240
MpHPPR    TNYTYYNSVVELASNSDILVVACALTPETTHIVNREVIDALGPKGVLINIGRGPHVDEAE 240
PvHPPR    TNYTYYGSVVELASNSDILVVACALTPETTHIVNRQVIDALGPKGILINIGRGPHIDEAE 240
          ******.**************** ********:**:*********:*********:** *

CbHPPR    LVSALVEGRLGGAGLDVFEREPEVPEKLFGLENVVLLPHVGSGTVETRKVMADLVVGNLE 300
MpHPPR    LVSALVEGRLGGAGLDVFEKEPEVPEQLFGLENVVLLPHVGSGTVETRKAMADLVLGNLE 300
PvHPPR    MVSALVEGRLGGAGLDVFEKEPEVPEQLFGLENVVLLPHVGSGTEETRKAMADLVLGNLE 300
          :******************:******:***************** ****.*****:****

CbHPPR    AHFSGKPLLTPVV      313
MpHPPR    AHFSSKPLLTPVV      313
PvHPPR    AHFSSKPLLTPVV      313
          ****.********
```

HPPR: hydroxyphenylpyruvic acid; CbHPPR: HPPR from *Coleus blumeii*; MpHPPR: HPPR from *Mentha* x *piperita* (*Mentha aquatica* x *Mentha spicata*); PvHPPR: HPPR from *Prunella vulgaris.* Ser53 - Asn54 in red (CbHPPR) // Ala53 - Thr54 in green

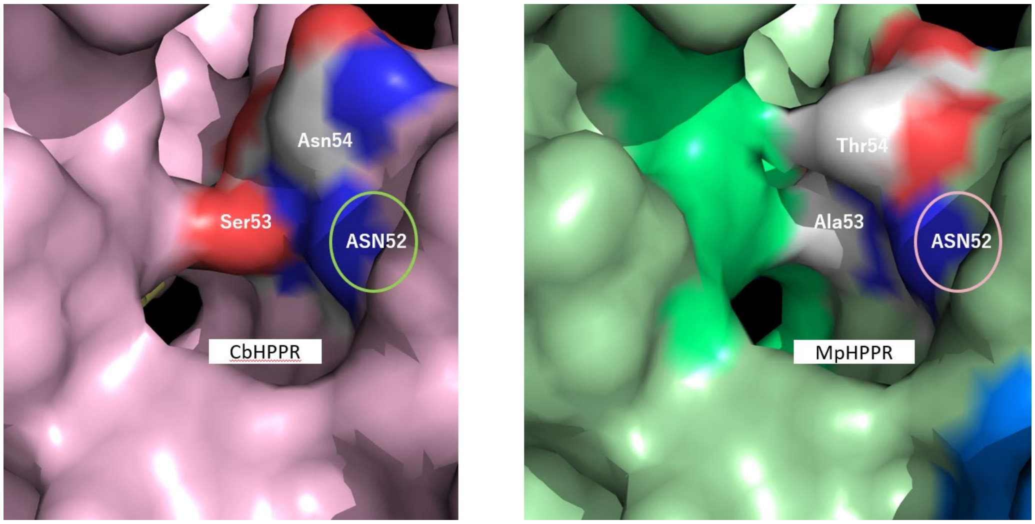

Figure S1. HPPRs sequence alignment and molecular visualization of CbHPPR and MpHPPR with Pymol (DeLano, 2002a). The amino acids residues in positions 53 and 54 are exhibited with the following colors: red for oxygen atom, blue for nitrogen atom and grey for the carbon atoms.

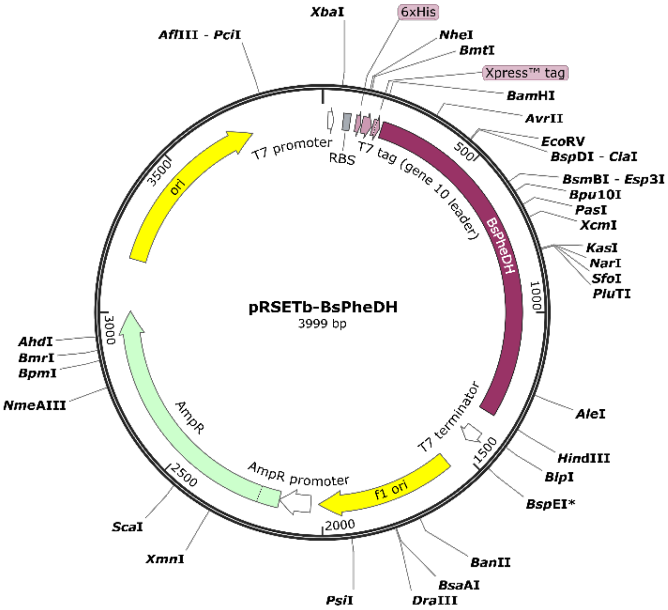

HHHHHHGMASMTGGQQMGRDLYDDDDKDPMAKQLEKSSKIGNEDVFQKIANHEQIVFCNDPVSGLQAIIAIHDTTLGPALGGTRMYPYKNVDEALEDVLRLSEGM
TYKCAAADIDFGGGKAVIIGDPEKDKSPALFRAFGQFVESLNGRFYTGTDMGTTMDDFVHAQKETNFINGIPEQYGGSGDSSIPTAQGVIYALKATNQYLFGSDSLSGK
TYAIQGLGKVGYKVAEQLLKAGADLFVTDIHENVLNSIKQKSEELGGSVTIVKSDDIYSVQADIFVPCAMGGIINDKTIPKLKVKAVVGSANNQLKDLRHANVLNEKGILY
APDYIVNAGGLIQVADELYGPNKERVLLKTKEIYRSLLEIFNQAALDCITTVEAANRKCQKTIEGQQTRNSFFSRGRRPKWNIKE

Figure S2. Plasmid map of pRSETb- BsPheDH and protein sequence of BsPheDH expressed with 6x(His)-tag in the N-terminal.

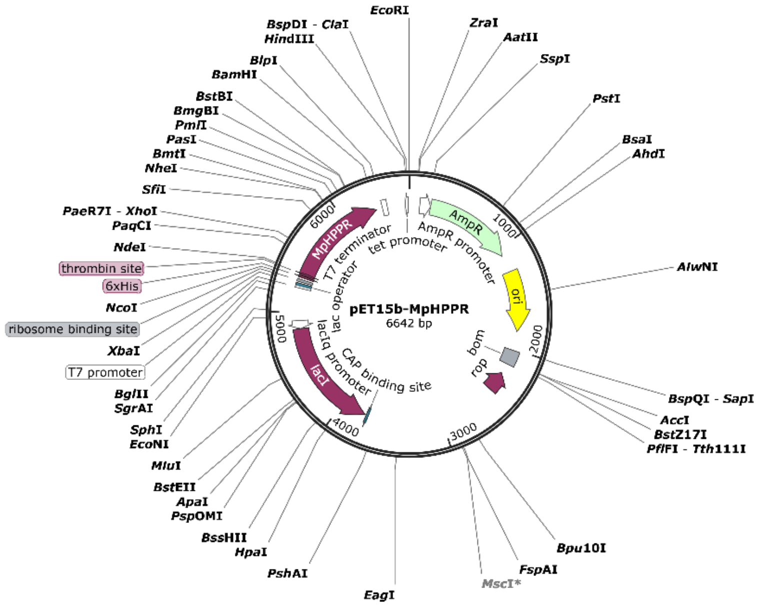

CATATGGAAGCTATAGGAGTACTAATGACATGTCCGATGAACAACTACCTGGAACAAGAACTGGACAAGCGCTTCAAGCTGTTCCGTTATTGGACCCAGCCGAA
GCAGCGTGAGTTCCTGGCGCAACACGCAGAAAGCATTCGCGCGGTCGTGGGAAACGCGACCGCAGGTGCGGATGCGGAGCTGATTGATGCGCTGCCGAAGCT
CGAGATCGTGTCGTCTTTTTCTGTAGGTTTAGACAAGGTGGACTTGAATAAATGCAAAGAGAAGGGTATTCGTGTTTCCAACACCCCGGACGTTTTGACCGATG
ATGTTGCCGACCTCGCGATCGGCCTTATGCTGGCGGTGTTGAGACGCATCTGCGAATGTGATAAGTATGTTCGTCGTGGTGCGTGGAAATTGGGCGATTTTAAG
CTGACGACCAAATTCAGCGGTAAGCGCGTTGGCATCCTGGGTTTAGGCCGTATCGGCCTGGCGGTTGCGGAACGTGCCGAGGCGTTTGATTGTCCGATTAGCTA
CTACAGCCGTAGTAAAAAAGCTAATACCAATTATACCTACTACAATTCCGTGGTCGAGTTGGCTAGCAACAGCGATATCTTGGTCGTGGCCTGCGCATTGACCCC
AGAGACTACGCATATTGTTAATCGTGAAGTGATCGACGCCCTGGGTCCGAAAGGTGTTCTGATCAACATTGGTCGTGGCCCTCACGTGGACGAAGCCGAGCTG
GTGAGCGCTCTGGTAGAGGGTCGCTTGGGCGGTGCTGGTCTGGACGTGTTCGAAAAAGAACCGGAAGTTCCGGAACAGCTGTTCGGCCTTGAGAACGTGGTG
TTGCTGCCGCACGTTGGTTCTGGCACCGTTGAGACGCGCAAAGCTATGGCAGACTTGGTTCTGGGCAACCTGGAGGCACATTTTTCCAGCAAACCGCTGCTGAC
TCCGGTTGTTTAA

HHHHHHSSGLVPRGSHMEAIGVLMTCPMNNYLEQELDKRFKLFRYWTQPKQREFLAQHAESIRAVVGNATAGADAELIDALPKLEIVSSFSVGLDKVDLNKCKEKGIR
VSNTPDVLTDDVADLAIGLMLAVLRRICECDKYVRRGAWKLGDFKLTTKFSGKRVGILGLGRIGLAVAERAEAFDCPISYYSRSKKANTNYTYYNSVVELASNSDILVVAC
ALTPETTHIVNREVIDALGPKGVLINIGRGPHVDEAELVSALVEGRLGGAGLDVFEKEPEVPEQLFGLENVVLLPHVGSGTVETRKAMADLVLGNLEAHFSSKPLLTPVV

Figure S3. Plasmid map of pET15b-MpHPPR, codon-optimized nucleotide sequence of the MpHPPR gene and protein sequence of MpHPPR expressed with 6x(His)-tag in the N-terminal.

2. Expression and purification of MpHPPR

The enzyme was overexpressed in 50 mL of LB or AI media at 37°C or 25°C, induced with 1 or 0.1 mM IPTG when LB media was used. An SDS-PAGE was run with the samples of the described expression conditions to compare the yield of insoluble/soluble proteins (Figure S4).

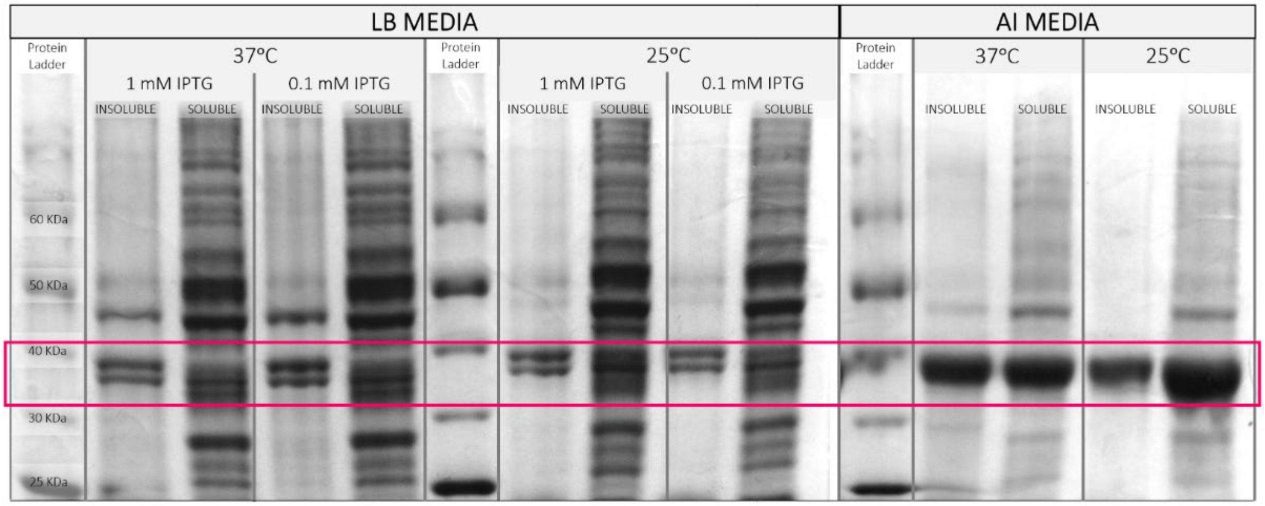

Figure S4. SDS-PAGE of expression conditions. The bands related to MpHPPR are located inside the pink rectangular.

Both insoluble and soluble fractions were found in all the tested conditions. However, the expression in autoinduction media at 25°C offered the highest amount of soluble overexpressed protein. Moreover, it gave the best ratio between soluble and insoluble proteins, where the soluble were predominant compared to the insoluble.

The activity of the crude extract was checked with DHPPA and NADH as substrates, and all the conditions seemed to give active proteins. The larger scale expression (AI - 25°C) produced 20 grams of pellet per liter of culture. Enzyme purification was performed by Nickel-affinity chromatography using a 5 mL His-trap column. A significant amount of MpHPPR was effectively purified, specifically 300 mg per liter of culture media, as shown in Figure S5.

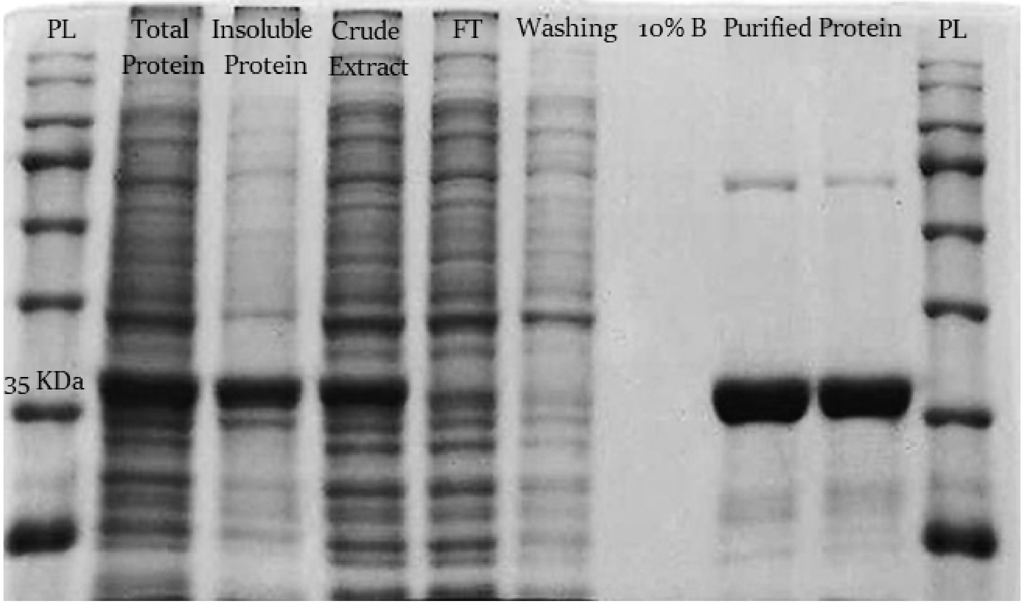

Figure S5. SDS-PAGE of MpHPPR purification process. PL: protein ladder. FT: flow-through.

3. Characterization of MpHPPR

As for the activity towards phenyl pyruvate, the results were very similar from pH 7 to pH 8 with slightly higher activity at pH 9 but still in the range considering the error (Figure S6). Different outcomes were found when using 4-hydroxyphenylpyruvic acid (HPPA) as substrate (Figure S7). The highest activity was achieved at pH 7.0 while increasing the pH revealed a faster oxidation rate of the substrate solution (visible brown color formation). Consequently, the lower activity obtained at higher pH may be caused by the reduced availability of the substrate, which was below the K_M. To avoid any error, all activity assays were performed at pH 7 in phosphate buffer.

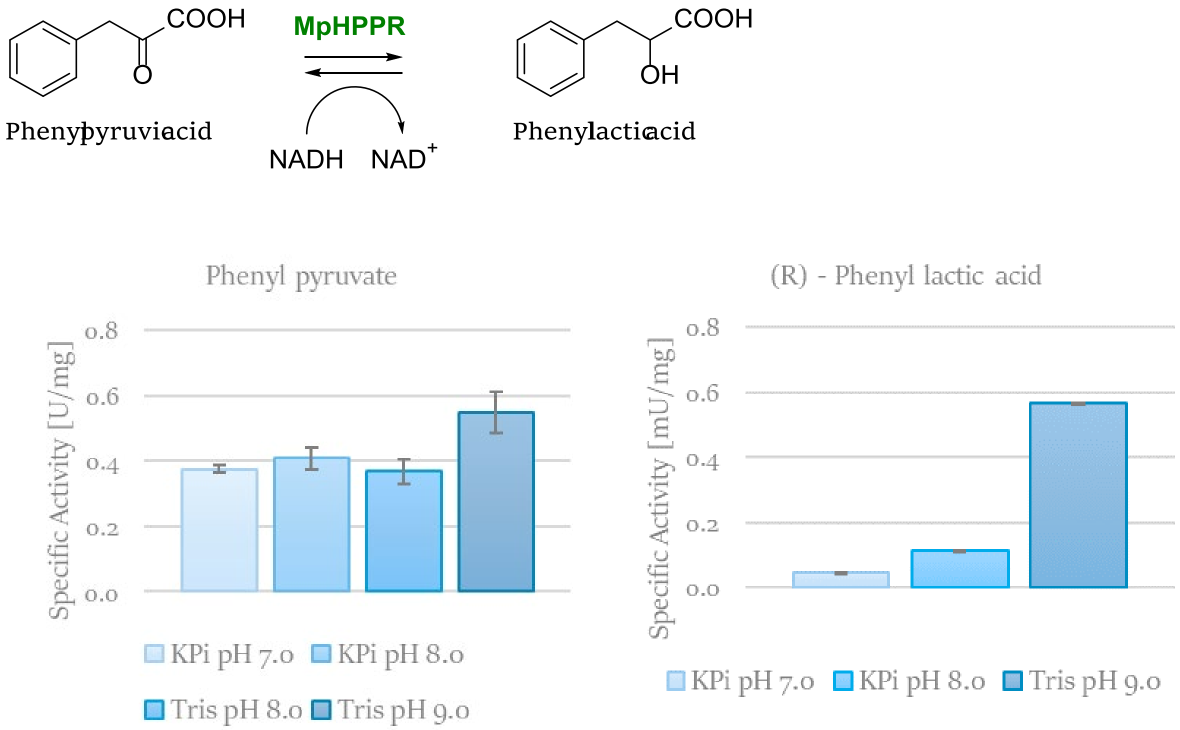

Figure S6. Scheme of reaction and specific activity of MpHPPR for phenylpyruvate and (R)-phenyl lactic acid. Temperature at 37°C, employing 30 mM substrate, 10 mM NAD(H), 5 mM ß-mercaptoethanol and the appropriate buffer: potassium phosphate (KPi) or Tris HCl buffer.

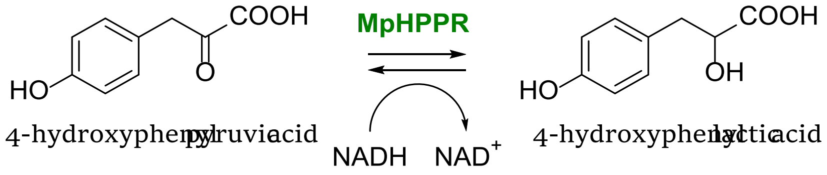

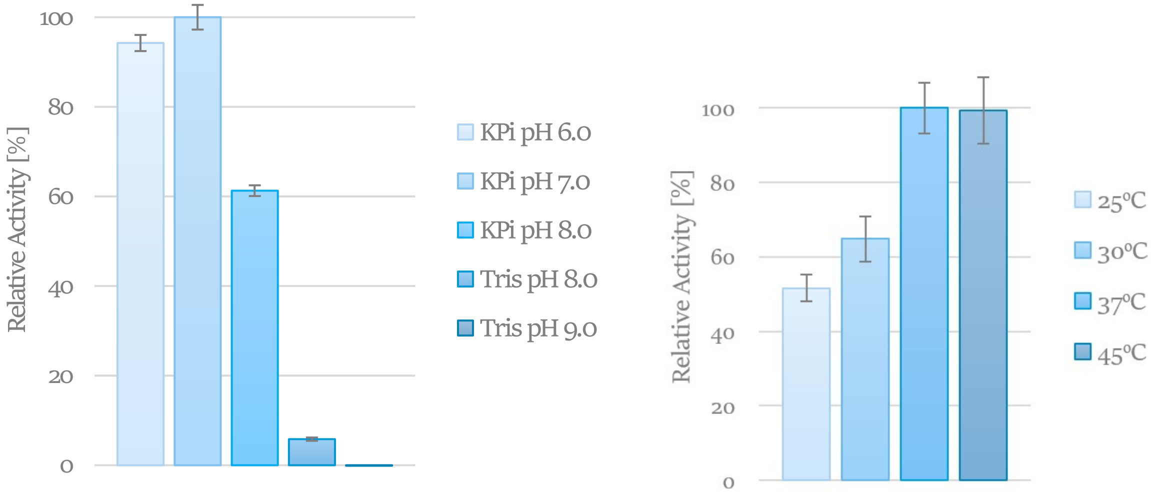

Figure S7. Scheme of reaction and specific activity of MpHPPR for HPPA. 30 mM substrate, 10 mM NADH, 5 mM ß-mercaptoethanol and the appropriate buffer: potassium phosphate (KPi) or Tris HCl buffer for the graph on the left (at 37°C), and KPi pH 7 over temperature for the graph on the right. Relative activity to pH 7 (left) and 37°C (right).

Table S1. Results of MpHPPR specific activity at different antioxidant additions using HPPA as substrate and NADH as cofactor in 50 mM potassium phosphate buffer pH 7 (37°C).

Antioxidant	Specific activity [U/mg]	Retained Activity [%]
/	0.44 ± 0.03	100
10 mM β-mercaptoethanol	0.43 ± 0.03	100
10 mM ascorbic acid	0.47 ± 0.03	110

4. Characterization of His-BsPheDH

The expression of His-BsPheDH yielded 16 g of pellet and 32 mg of purified enzyme (per L of culture).

Table S2. Specific activity of His-BsPheDH at different buffer conditions using 10 mM L-phenylalanine and 2.5 mM NAD^+ as substrates. All the buffers were tested at a concentration of 50 mM. The reaction is triggered with 10 µL of protein solution (stock of 0.004 – 0.001 mg/mL) in 400 µL of total reaction volume. Absorbance at 340 nm (ε: 6.22 mM^{-1} cm^{-1}), monitored for 2 minutes at 25°C. Activity tests are performed in triplicates.

Buffer	pH	Antioxidant	Specific activity [U/mg]	Retained Activity [%]
Glycine NaOH	10.4	/	120 ± 9	100
Tris HCl	8.5	/	20.0 ± 2.5	17
	8.0	/	16.4 ± 1.9	14
Potassium Phosphate	8.0	/	13.0 ± 2.5	11
	7.0	/	2.0 ± 0.2	2

Glycine NaOH	10.4	10 mM β-mercaptoethanol	78 ± 3	65
		20 mM β-mercaptoethanol	69 ± 9	58
		10 mM ascorbic acid	110 ± 5	92
		20 mM ascorbic acid	33 ± 3	28

5. Immobilization screening of His-BsPheDH and MpHPPR

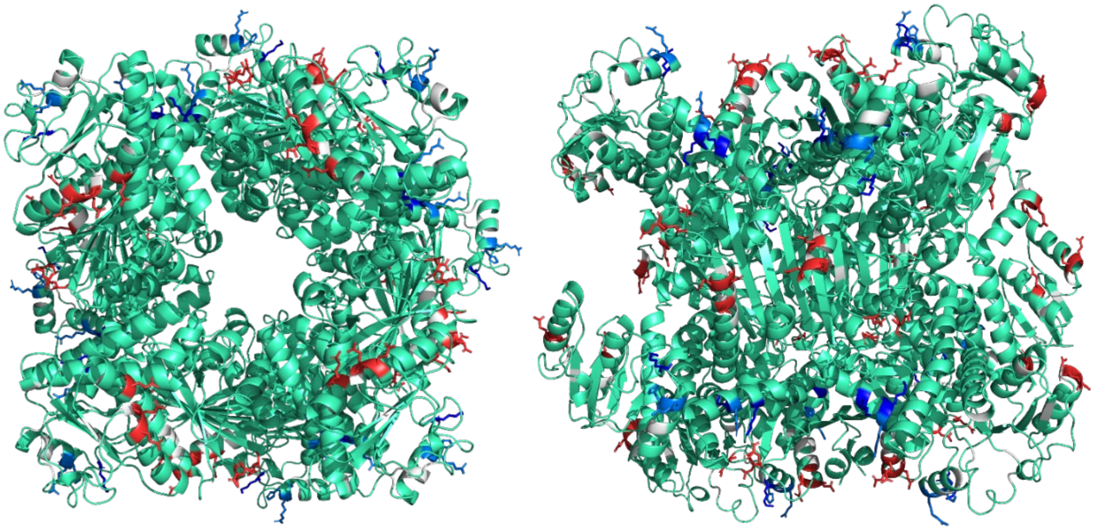

Figure S8. His-BsPheDH structure analyzed by CapiPy tool (Roura Padrosa et al. 2021). Graphic visualization in Pymol, image taken from the top and from the side of the octamer (DeLano, 2002a). In blue: Lys, in red: Asp/Glu.

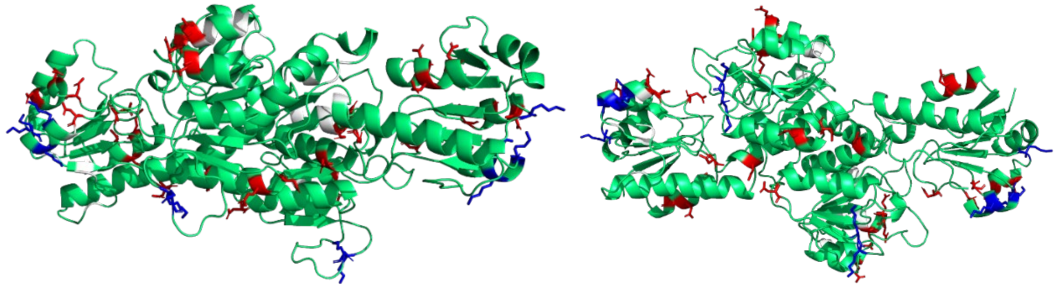

Figure S9. MpHPPR structure analyzed by CapiPy tool (Roura Padrosa et al. 2021). Graphic visualization in Pymol, images taken from the front and back sides of the dimer (DeLano, 2002a). In blue: Lys, in red: Asp/Glu, white: hydrophobic residues.

His-BsPheDH was immobilized onto the methacrylate resin EP400/SS using different strategies (Table S3).

Table S3. Results of His-BsPheDH immobilization on EP400/SS with a protein loading of 1 mg/g.

Functional Groups – Immob. Strategy	Immobilization Yield [%]	Recovered Activity [%]

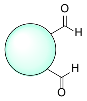	Aldehydes - covalent	99	27 ± 3
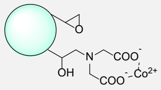	Epoxy/Cobalt - oriented and covalent	>99	36 ± 2
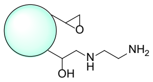	Epoxy/Amino - oriented and covalent	>99	26 ± 3
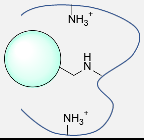	Amino - ionic interaction	>99	92 ± 5

Two loading capacities were tested to ensure a sufficient level of activity of MpHPPR (Table S4). Indeed, the low activity recovered after immobilization of 1 mg/g on epoxy and amino resin was not easy to assess, while a 5 times higher loading provided a more precise outcome. In any case, the activity was too low to be considered as strategy. The same was observed for the covalent immobilization on glyoxyl groups. In fact, the enzyme was killed after the incubation step with $NaBH_4$ and no activity was detected even with higher loading.

Table S4. Results of MpHPPR immobilization on EP400/SS.

Functional Groups – Immob. strategy		MpHPPR Loading [mg/g]	Immobilization Yield [%]	Recovered Activity [%]
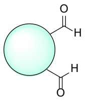	Aldehydes - covalent	1	>99	0
		5		
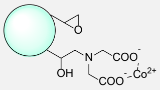	Epoxy/Cobalt - oriented covalent	1	>99	39 ± 1
		5	>99	39 ± 1
		40	>99	37 ± 1
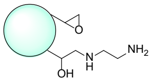	Epoxy/Amino - oriented covalent	1	70	<1
		5	70	7 ± 2
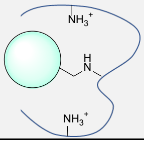	Amino - ionic interaction	1	>99	83 ± 1
		5	>99	89 ± 2

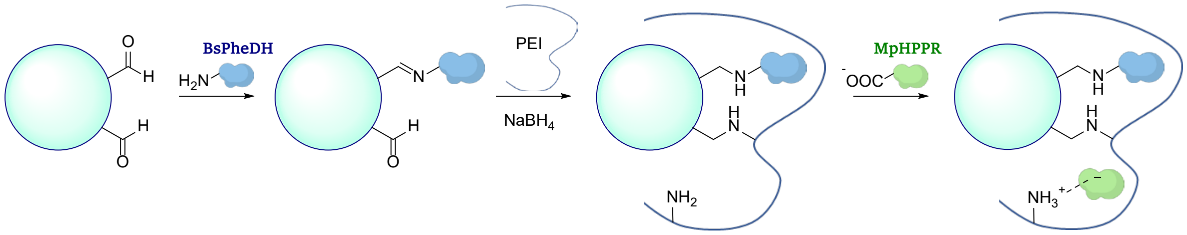

Figure S10. Co-immobilization by first interaction of His-BsPheDH with aldehydes, then coated with PEI followed by reduction, and last interaction of MpHPPR with PEI.

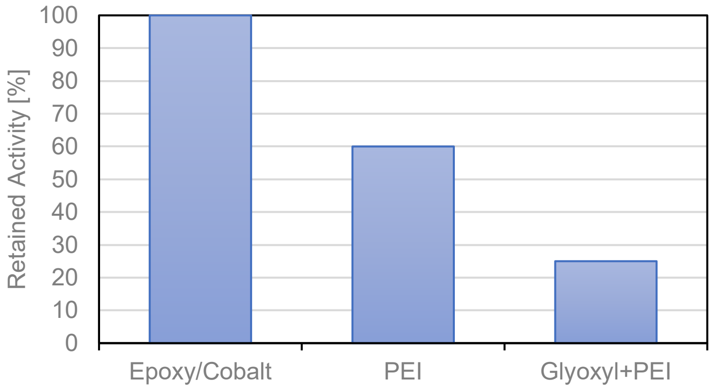

Figure S11. Operational stability after 6 cycles of **2-hours** biotransformation with co-immobilized enzymes.

With the sequential immobilization on Glyoxyl and PEI, the enzyme activity was rapidly dropped within the third cycle, due to MpHPPR leaching (ionic interactions are not as stable as the covalent ones). Therefore, the cross-linking of MpHPPR with glutaraldehyde (GA) was trialed to covalently stabilize the enzyme on the support and avoid any leaching in working conditions. The protein was cross-linked without the reduction step since it was known to be detrimental for the enzyme stability. However, MpHPPR retained only 8% of the initial recovered activity after the treatment with GA. Given the unsuccessful outcome, this immobilization strategy was not considered.

6. Optimization of protein immobilization

More tests were performed to improve the His-BsPheDH immobilization:

- Immobilization on PEI, then incubation with GA to form stable bonds → only 28% activity was retained after GA treatment
- 0.5 mg/mL of PEI with protein during immobilization on epoxy/cobalt (for protein stabilization) → no activity was recovered
- Glyoxyl immobilization at pH 8.5 instead of pH 10 → no improvement was obtained
- DVS immobilization at pH 7 or pH 10 → no improvement compared to epoxy/cobalt
- More tested supports → S2200-PC from Evonik, EC-HFA/S and HFA403/S from Resindion, but the recovered activity was less than half compared to the support EP400/SS with His-BsPheDH (immobilization strategies: glyoxyl, epoxides and cobalt, epoxides and amino groups). Instead, EP403/S from Resindion achieved almost similar results to EP400/SS. Since no

improvement was obtained with these four similar supports, they were not considered. Instead, the polysaccharide matrix agarose showed similar results to EP400/SS while possessing different characteristics compared to the methacrylate resins, so it was used in the optimization process as comparison.

Table S5. Optimization of His-BsPheDH immobilization.

Loading	Strategy	Support	Incubation Time	Immobilization Yield	Recovered Activity
2.5 mg/g	Epoxy/cobalt	EP400/SS	8 h	> 99 %	12 ± 2 %
		Ag 6% BCL		> 99 %	11 ± 0.5 %
5.0 mg/g	Epoxy/cobalt with Gly as blocking agent	Ag 6% BCL	8 h	> 99 %	8 ± 0.4 %
		EP400/SS		> 99 %	8 ± 0.4 %
		Ag 6% BCL	24 h	> 99 %	7 ± 0.8 %
		EP400/SS		> 99 %	7 ± 0.8 %
	Epoxy/cobalt with EDA as blocking agent	Ag 6% BCL	24 h	> 99 %	8 ± 0.8 %

Table S6. Optimization of MpHPPR immobilization with a protein loading of 40 mg/g.

Strategy	Support	Incubation Time	Immobilization Yield	Recovered Activity
Epoxy/cobalt with Gly as blocking agent	Ag 6% BCL	8 h	85 %	36 ± 1 %
	EP400/SS		> 99 %	32 ± 1 %
	Ag 6% BCL	24 h	> 99 %	66 ± 2 %
	EP400/SS		> 99 %	41 ± 1 %
Epoxy/cobalt with EDA as blocking agent	Ag 6% BCL	24 h	> 99 %	60 ± 6 %

Table S7. Optimization of co-immobilization using epoxy/cobalt as strategy with a protein loading of 2.5 mg/g for His-BsPheDH and 40 mg/g for MpHPPR.

Strategy	Incubation Time	Support	Yield of MpHPPR	Yield of BsPheDH	Total Protein Yield
Sequential	8 h	Ag 6% BCL	86 %	36 %	/
		EP400/SS	93 %	> 99 %	/
	24 h	Ag 6% BCL	99 %	98 %	/
Simultaneous	8 h	Ag 6% BCL	/	/	82 %
		EP400/SS	/	/	97 %
	24 h	Ag 6% BCL	/	/	84 %
		EP400/SS	/	/	> 99 %

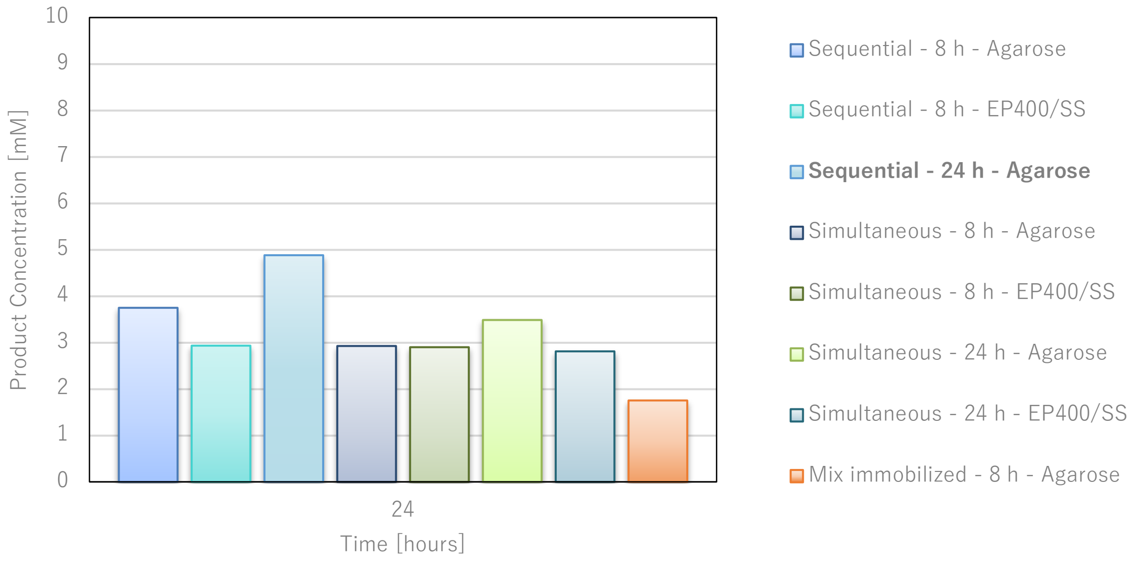

Figure S12. Biotransformation with 100 mg of co-immobilized MpHPPR and His-BsPheDH. Conditions: 10 mM L-dopa, 10 mM ascorbic acid, 1 mM NAD^+ in 50 mM TrisHCl pH 8.0. Total volume: 1 mL. Incubation at 37°C and 150 rpm.

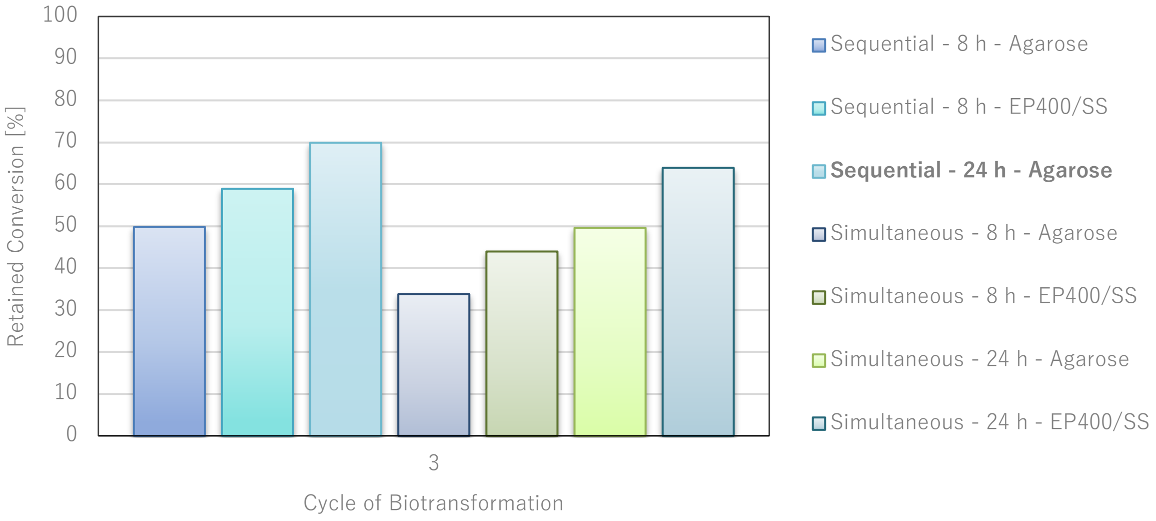

Figure S13. Operational stability after 3 cycles of biotransformation with 100 mg of MpHPPR and His-BsPheDH co-immobilized with the strategies in Table S7. Conditions: 10 mM L-dopa, 10 mM ascorbic acid, 1 mM NAD^+ in 50 mM TrisHCl pH 8.0. Total volume: 1 mL. Incubation at 37°C and 150 rpm.

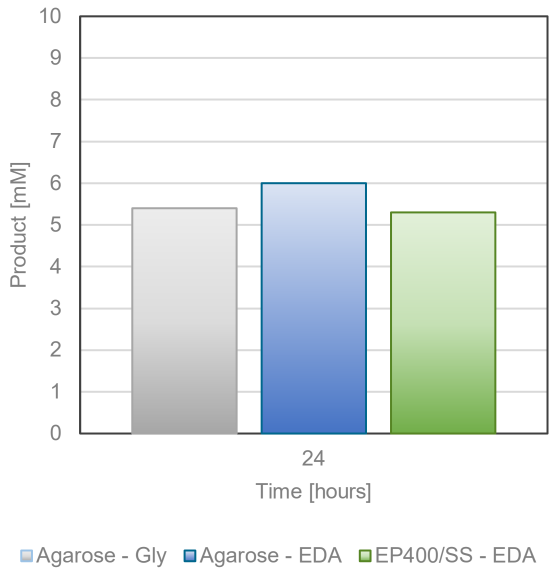

a)

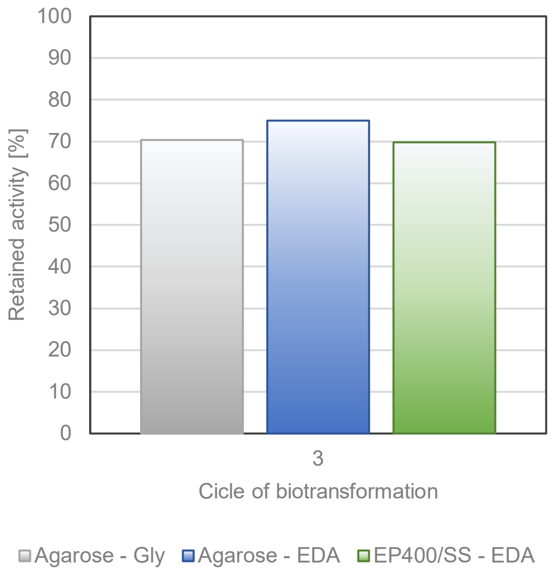

b)

Figure S14. (a) Product formation after 24 hours of first biotransformation. (b) Operational stability after 3 cycles of biotransformation with 100 mg of co-immobilized MpHPPR and His-BsPheDH, having different blocking agents. Conditions: 10 mM L-dopa, 10 mM ascorbic acid, 1 mM NAD^+ in 50 mM TrisHCl pH 8.0. Total volume: 1 mL. Incubation at 37°C and 150 rpm.

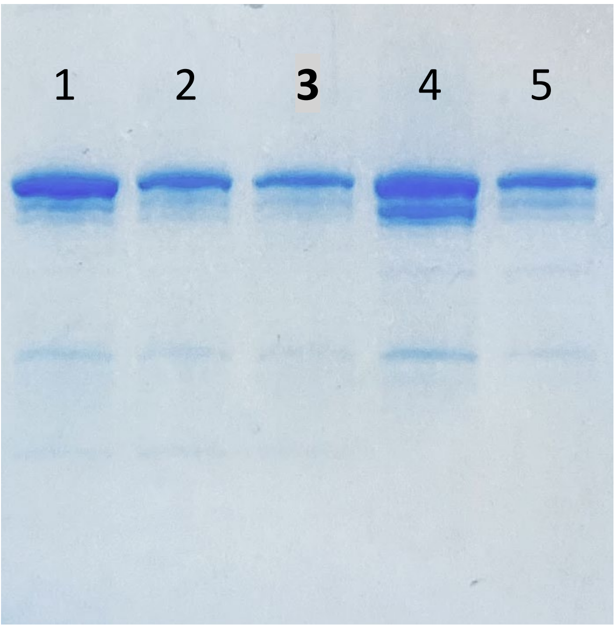

Figure S15. SDS-PAGE of boiled resin after His-BsPheDH immobilization (5 mg/g). 1: 8 hours incubation on agarose, glycine as blocking agent; 2: 24 hours incubation on agarose, glycine as blocking agent; 3: 24 hours incubation on agarose, EDA as blocking agent; 4: 8 hours incubation on EP400/SS, glycine as blocking agent; 5: 24 hours incubation on EP400/SS, EDA as blocking agent.

7. Characterization of danshensu

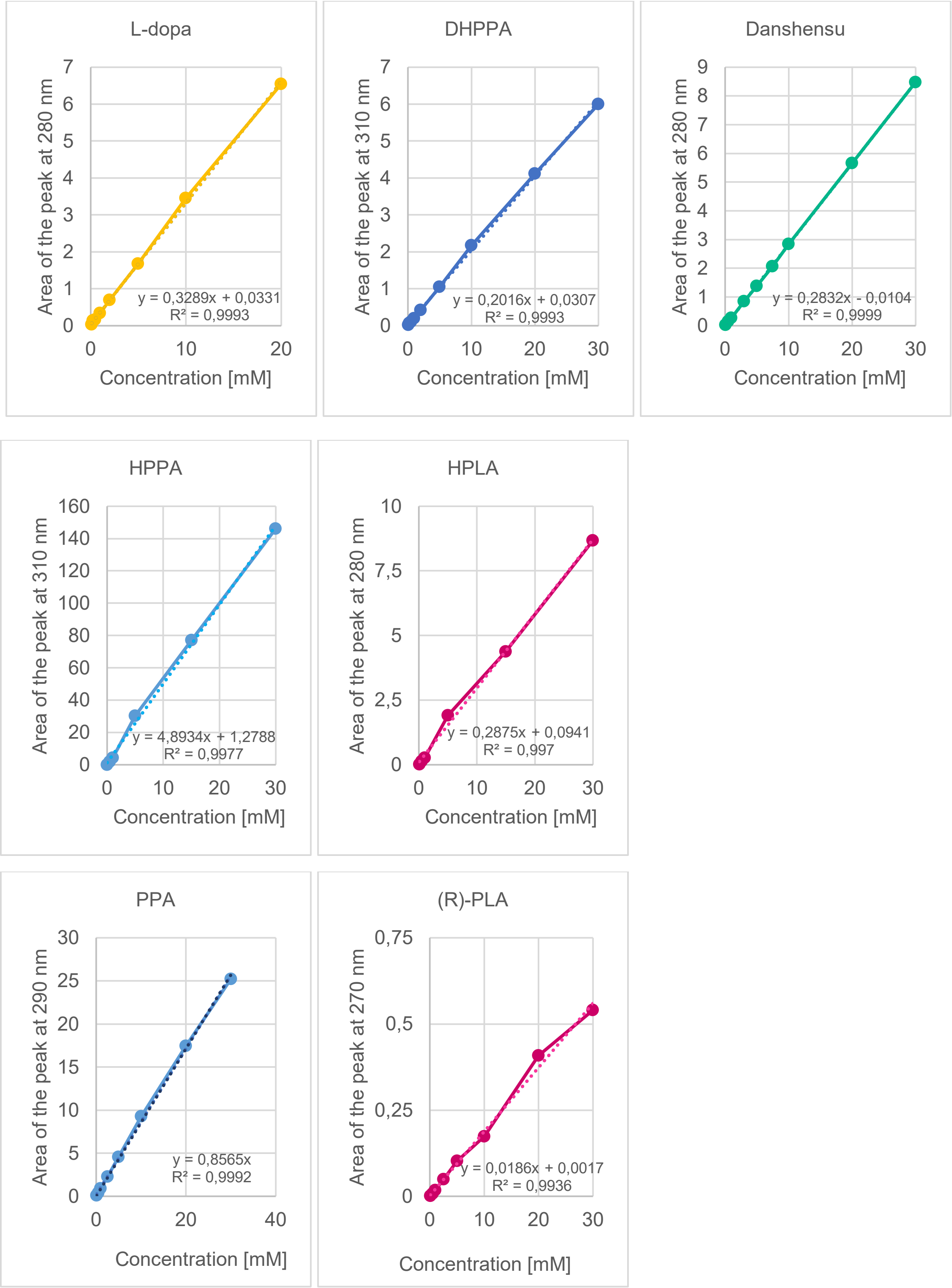

Figure S16. Calibration curves of standard compounds run in the HPLC.

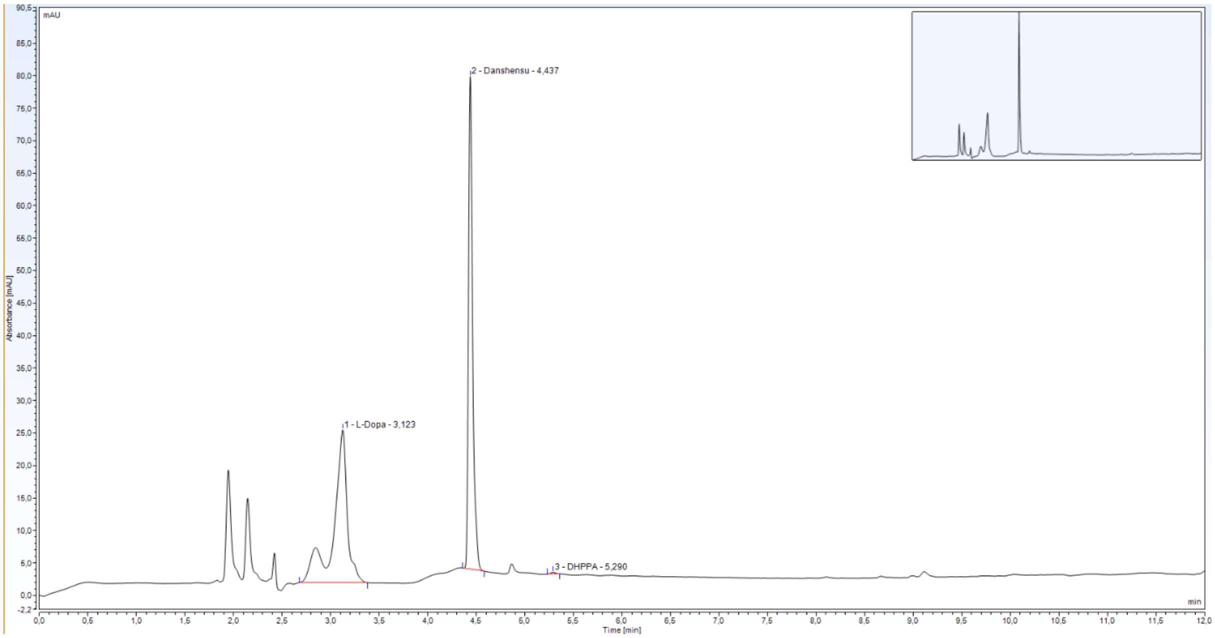

Figure S17. HPLC chromatography of a sample taken from continuous flow biotransformation (CV4, 60 min residence time).

Initially, β-mercaptoethanol was employed as antioxidant in the biotransformations. However, β-mercaptoethanol was extracted with danshensu, and the final purity of the dry sample was only 47% (53% of antioxidant, see Figure S19). For this reason, β-mercaptoethanol was not used in this project and it was replaced by ascorbic acid.

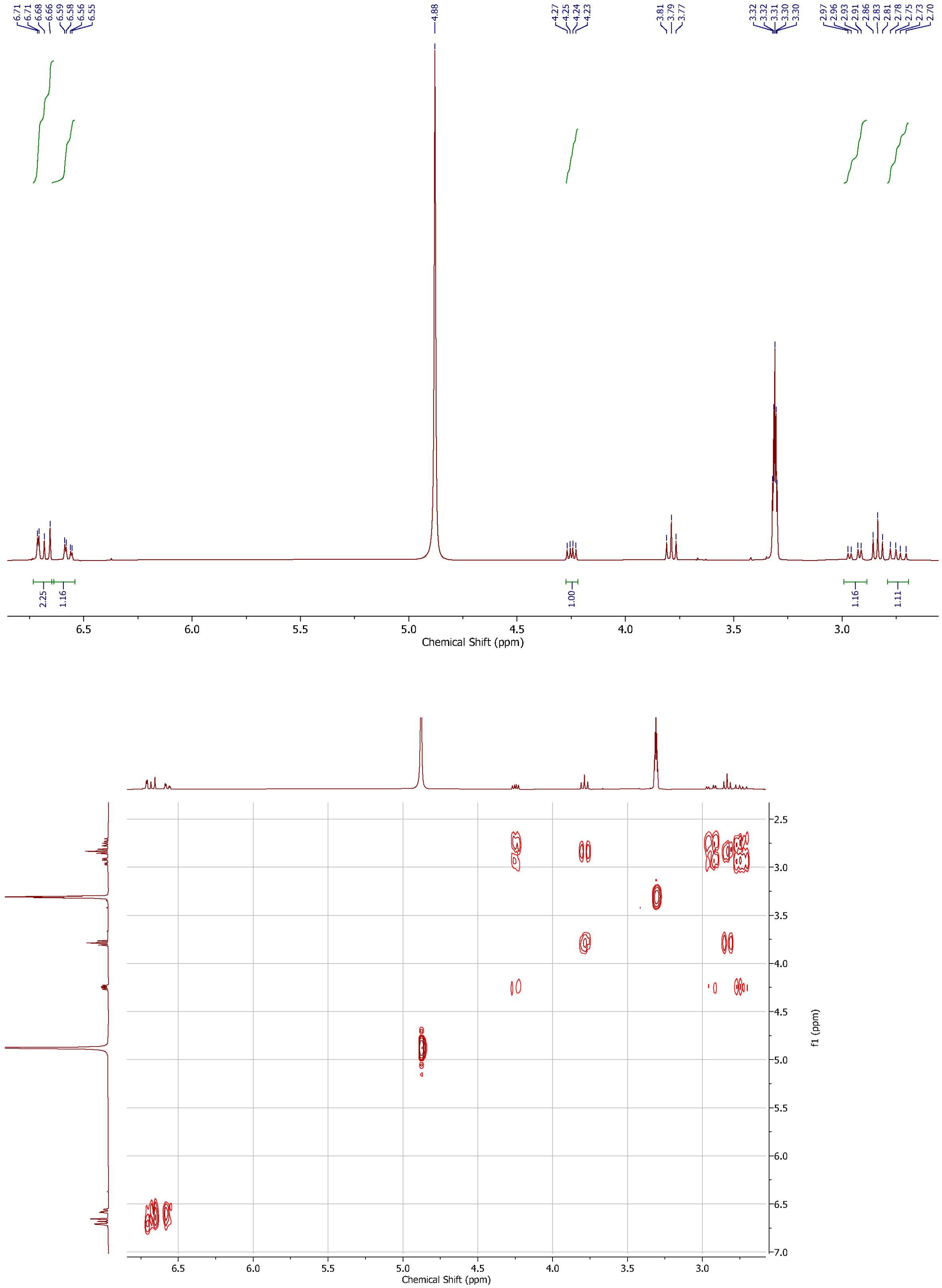

Figure S18. ^{1}H-NMR and COSY spectra of dry extracted product by ethyl acetate, when β-mercaptoethanol was tested as antioxidant.

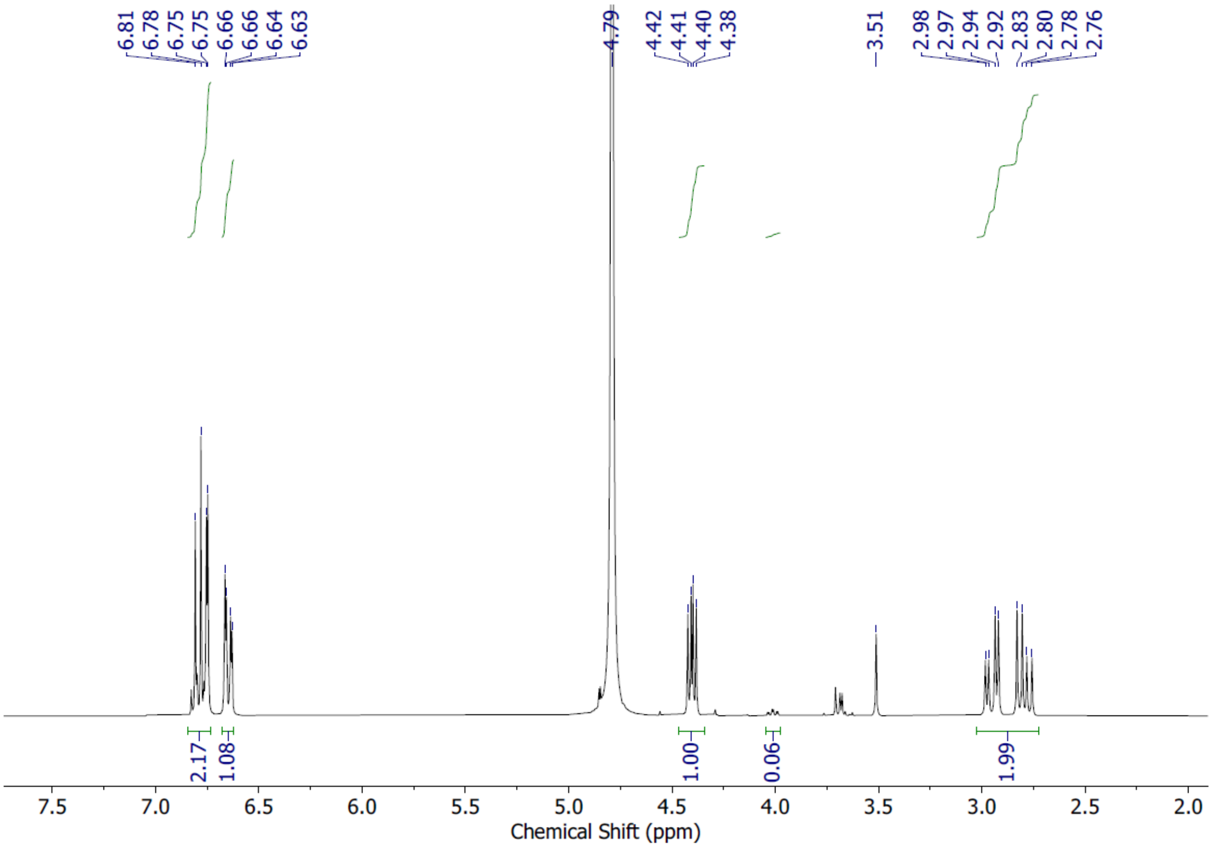

Figure S19. ^{1}H-NMR spectra of dry extracted product formed in continuous flow reaction.

8. Supplementary References

DeLano WL (2002) Pymol: An open-source molecular graphics tool. CCP4 Newsletter on protein crystallography 40:82–92

Guisán JM (1988) Aldehyde-agarose gels as activated supports for immobilization-stabilization of enzymes. Enzyme Microb Technol 10:375–382. https://doi.org/10.1016/0141-0229(88)90018-X

Mateo C, Abian O, Fernandez-Lafuente R, Guisan JM (2000) Reversible enzyme immobilization via a very strong and nondistorting ionic adsorption on support-polyethylenimine composites. Biotechnol Bioeng 68:98–105. https://doi.org/10.1002/(SICI)1097-0290(20000405)68:1<98::AID-BIT12>3.0.CO;2-T

Mateo C, Palomo JM, Fernandez-Lorente G, Guisan JM, Fernandez-Lafuente R (2007) Improvement of enzyme activity, stability and selectivity via immobilization techniques. Enzyme Microb Technol 40:1451–1463. https://doi.org/10.1016/j.enzmictec.2007.01.018

Roura Padrosa D, Marchini V, Paradisi F (2021) CapiPy: Python-based GUI-application to assist in protein immobilization. Bioinformatics 37:2761–2762. https://doi.org/10.1093/bioinformatics/btab030

Trobo-Maseda L, Orrego AH, Romero-Fernández M, Guisan JM, Rocha-Martín J (2020) Immobilization of Enzymes on Hetero-Functional Supports: Physical Adsorption Plus Additional Covalent Immobilization. Methods Mol Biol 2100:159–174. https://doi.org/10.1007/978-1-0716-0215-7_10

Velasco-Lozano S, Benítez-Mateos AI, López-Gallego F (2017) Co-immobilized Phosphorylated Cofactors and Enzymes as Self-Sufficient Heterogeneous Biocatalysts for Chemical Processes. Angew Chem Int Ed 56:771–775. https://doi.org/10.1002/anie.201609758
